# Controlled Debundling of Single-Walled Carbon Nanotubes (SWCNTs) by Au@Pt Nanorods Enables Mechanism-Dependent Electrochemical Sensing and Biofouling Response

**DOI:** 10.1021/acs.analchem.6c00924

**Published:** 2026-07-14

**Authors:** Bahar Mostafiz, Emil Rosqvist, Ermei Mäkilä, Vipul Sharma, Emilia Peltola

**Affiliations:** † Department of Mechanical and Materials Engineering, 8058 University of Turku, Turku FI-20014, Finland; ‡ Laboratory of Molecular Science and Engineering, 1040 Åbo Akademi University, Henriksgatan 2, Åbo FI-20500, Finland; § Department of Physics and Astronomy, University of Turku, Turku FI-20014, Finland

## Abstract

Single-walled carbon nanotubes (SWCNTs) form intrinsically bundled networks due to strong intertube interactions, yet conventional debundling approaches can disrupt or chemically alter the nanotube structure. Here, Au@Pt nanorods (NRs) were progressively incorporated into SWCNT films as a nondestructive strategy to deliberately debundle the network while preserving the carbon framework and introducing Pt-rich catalytic sites. This approach was used to examine how network restructuring and metal decoration govern biofouling and electrochemical sensing. Increasing NR loading reorganized the SWCNT network into thinner strands, changed conductive pathways, and increased accessible surface features and hydrophilicity. These changes yielded analyte-dependent electrochemical responses: dopamine (DA) oxidation became more adsorption-controlled after debundling, with enhanced faradaic and capacitive currents attributed to improved interfacial accumulation at carbon-rich surfaces, whereas hydrogen peroxide (H_2_O_2_) oxidation was dominated by Pt-mediated catalysis and increased with NR loading due to higher catalytic site density. Biofouling studies with bovine serum albumin (BSA) showed that high NR contents promoted protein adsorption and suppressed electrochemical activity. Interestingly, DA oxidation was least affected on pristine SWCNT electrodes, whereas H_2_O_2_ detection benefited from intermediate NR decoration, indicating that biofouling can be mitigated by tailoring the platform to the target analyte to maintain performance after protein exposure, rather than relying on antifouling surfaces.

## Graphical abstract

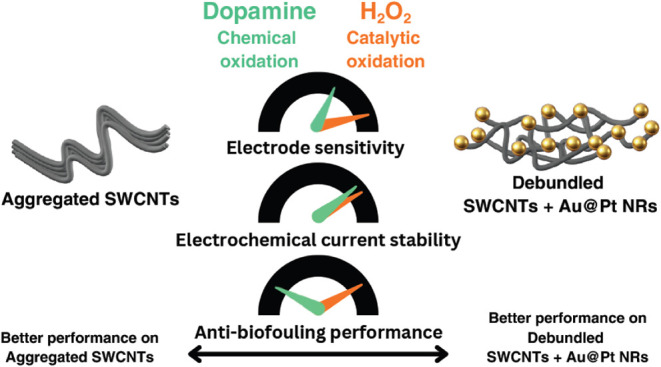

## Introduction

Neurotransmitters regulate neural communication, cognition, behavior, and motor function; therefore, their dysregulation is closely linked to neurodegenerative diseases such as Parkinson’s and Alzheimer’s disease.^1,2^ Efficient monitoring of neurotransmitter dynamics is thus important for diagnosis and prognosis. Dopamine (DA), a key catecholaminergic neurotransmitter, and hydrogen peroxide (H_2_O_2_), a byproduct of glutamate (another major neurotransmitter) oxidation, provide complementary information on neurotransmission and the local biochemical environment.

In the brain extracellular space, DA is typically present at basal concentrations of a few to tens of nanomolar, which can briefly rise into the hundreds of nanomolar range during stimulation.^3–5^ These low concentrations highlight the need for highly sensitive analytical platforms for DA monitoring. DA undergoes a two-electron, two-proton oxidation at its catechol group to form dopaminequinone (DAQ), making its electrochemical response strongly dependent on the electrode surface structure and chemistry.^6^

H_2_O_2_ is a small, redox-active molecule involved in many metabolic pathways^7^ and can be produced enzymatically by glutamate oxidase. In biological fluids such as plasma, normal concentrations are generally in the low micromolar range (1–5 μM), but may rise to approximately 50 μM^8^ during inflammation or oxidative stress. Measuring H_2_O_2_ in the body is challenging because its concentrations can vary greatly between locations and it decomposes rapidly.^9^

Electrochemical methods, particularly voltammetric techniques, are well suited for DA and H_2_O_2_ detection, because they rely on direct analyte oxidation or reduction at the electrode surface. They require small sample volumes, provide good temporal resolution and sensitivity, and can be miniaturized into flexible or implantable formats for monitoring neurochemical changes in biologically relevant environments.

Carbon materials are widely used as electrode substrates because they are inexpensive, conductive, chemically stable, and surface-modifiable. Single-walled carbon nanotubes (SWCNTs) are especially promising. They are essentially rolled-up graphene sheets with nanometer-scale diameters and very high aspect ratios, which give them excellent electrical properties and a large accessible surface area. They can be found in varying configurations depending on how densely individual tubes clump together, forming aggregates. Their aggregation density may strongly influence film electrochemical performance, particularly for analytes with different redox mechanisms. SWCNTs can form thin, flexible films and composites compatible with soft tissues.^10^ They can promote direct electron transfer between redox-active sites and the electrode, acting as efficient nanoscale “electron highways”.^11^ Compared to some types of multiwalled carbon nanotubes (MWCNTs), which have raised toxicity and carcinogenicity concerns, SWCNTs are generally safer under relevant exposure conditions, supporting their use in biomedical sensors.^12^

Metal nanoparticles are often incorporated into electrodes to enhance electrochemical performance. Gold (Au) and platinum (Pt) nanoparticles provide abundant catalytic sites for reactions (e.g., H_2_O_2_ oxidation), while minimizing metal loading. Their small size also helps them integrate better with soft and flexible supports. Core–shell nanorods (NRs) introduce an additional level of control: the core and shell can be composed of different materials, enabling precise tuning of stability, surface chemistry, and, crucially, size distribution; a parameter that strongly governs NRs’ behavior. In principle, this allows simultaneous optimization of both catalytic activity and biocompatibility. Therefore, it is essential for rational sensor design to understand how and where these particles modify the local surface chemistry and charge-transfer processes and how such NRs interact with SWCNTs.^13–15^

Many studies have examined (a) nanoparticle electroplating, electrodeposition, or attachment onto surface functionalized carbon nanotubes (CNTs), or (b) CNTs growth using metallic nanoparticles as catalysts or nucleation sites.^16,17^ However, important gaps remain regarding how metallic nanoparticles in the nanotube environment affect (a) the charge environment in intertube regions, (b) the regions they likely occupy within the tube structure, (c) the defect and edge sites introduced by their presence, and (d) the behavior of these regions when the electrode surface is exposed to target molecules with intrinsically different oxidation mechanisms. Understanding these effects can guide more effective SWCNT functionalization and modification, particularly for health technology device manufacturing.

Electrochemical sensors used in biological media are susceptible to biofouling, as proteins and other biomolecules rapidly adsorb onto the electrode and alter the local charge, hydration, and accessibility of active sites.^18^ This reduces sensitivity and worsens detection limits, which is especially problematic for low-concentration analytes such as DA and H_2_O_2_. Tuning SWCNT properties may help mitigate these effects and improve sensor durability.

Here, we address these challenges by integrating Au@Pt NRs with flexible SWCNTs films. By systematically increasing NRs loading and consequently reducing the SWCNTs aggregates’ size, we examine changes in surface chemistry, electrochemical behavior, and biofouling resistance. This establishes a more rational strategy for incorporating CNTs into sensor fabrication.

## Experimental Section

### Reagents

The materials needed for making the SWCNTs solution were SWCNTs soot (01RW03.N1, TUBALL), and sodium deoxycholate (DOC). The synthesis of Au@Pt NRs involved: cetyltrimethylammonium bromide (CTAB), gold­(III) chloride trihydrate (HAuCl_4_·3H_2_O), silver nitrate (AgNO_3_), sodium chloride (NaCl), l-ascorbic acid (AA), sodium borohydride (NaBH_4_), polyvinylpyrrolindone (PVP), sodium hydroxide (NaOH), and potassium tetrachloroplatinate­(II) (K_2_PtCl_4_), all dissolved in ultrapure water.

To prepare a phosphate buffer saline (PBS) with a final pH of 7.4, salt (NaCl), disodium hydrogen phosphate (Na_2_HPO_4_), potassium chloride (KCl), and monopotassium phosphate (KH_2_PO_4_) were dissolved in ultrapure water. Electrochemical studies were carried out by making DA hydrochloride (4-(2-aminoethyl)­benzene-1,2-diol hydrochloride 98%) solution in PBS and H_2_O_2_, sulfuric acid 96% (H_2_SO_4_), hexaamineruthenium­(III) chloride ([Ru­(NH_3_)_6_]­Cl_3_), and hexacyanoferrate­(III) [Fe­(CN)_6_]^−3^ solutions in ultrapure water. For protein incubation steps, bovine serum albumin (BSA) was dissolved in PBS.

Except for KCl and KH_2_PO_4_ (VWR), SWCNTs (OCSiAI, Luxembourg), and DOC (BioChemica), all chemicals were obtained from Sigma-Aldrich.

### Apparatus

Electrochemical measurements were performed utilizing a Gamry Reference 620 (Warminster, USA) potentiostat in a glass cell with a 3-electrode setup. The reference electrode was Ag/AgCl (CH Instruments), the counter electrode was a Pt wire, and the working electrode was fabricated in-house.

Transmission electron microscopy (TEM) was conducted on a Jeol JEM-1400Plus TEM operating at 80 kV.

Scanning electron microscopy (SEM) and Energy-dispersive X-ray spectroscopy (EDS) imaging were carried out using a Thermo Scientific Apreo S field-emission SEM equipped with an UltimMax 100 EDS detector (Oxford Instruments). The electron micrographs were obtained using an acceleration voltage of 2 kV, while the EDS was done with an acceleration voltage of 4 kV.

Atomic force microscopy (AFM) was used in surface characterization of samples before and after protein incubation. Bruker Multimode 8 AFM (Bruker, CA) was operated in PeakForce mode. FMV-A cantilevers (nominal spring constant k = 1–5 N/m and nominal radius of curvature r = 6 nm) were used (Bruker, CA). The cantilever spring constants were obtained through the thermal tuning method, and tip radius calibration was carried out using the relative approach (the radius was adjusted to match the known stiffness modulus of a reference sample). Conductive atomic force microscopy (c-AFM) measurements were carried out using a MultiMode 8 AFM system coupled with a Nanoscope V controller (Bruker, CA). The probes used were SCM-PIC-V2 antimony-doped silicon (Bruker, CA) coated with Pt–Ir (nominal spring constant k = 0.10 N/m and nominal radius of curvature r = 25 nm). Scans were collected at a rate of 2 Hz, employing a current sensitivity of 100 nA V^–1^. Several DC bias voltages (−100, 0, 100, 200, and 300 mV) were applied during imaging. Imaging was conducted at room temperature (RT) (23 ± 2° C) and a relative humidity of 20 ± 3%.

Equilibrium Contact Angles (ECA) and Surface Free Energy (SFE) measurements were obtained for each platform using 2 μL droplets of ultrapure water and ethylene glycol (EG), respectively, and 1 μL droplets of diiodomethane (DIM). Droplets were recorded over a 5 s interval using a CAM 200 optical contact angle meter equipped with a CCD camera (10 fps, KSV Instruments Ltd., Finland). All measurements were performed at RT and a relative humidity of 20 ± 3%.

Resistivity, sheet resistance, and conductivity of the prepared working electrodes were determined using an Ossila four-point probe system.

X-ray photoelectron spectra (XPS) were collected using Thermo Scientific Nexsa surface spectrometer with monochromatic Al Kα source (1486.7 eV). All the spectra were acquired with a spot size of 400 μm, and dual-beam charge compensation was applied.

### Particle Fabrication and Electrode Modification Procedure

The procedure for Au@Pt core–shell NRs fabrication is based on a method outlined in our previous studies;^9,19^ a full description can be found in the SI file.

To fabricate the electrodes, the SWCNTs solution was prepared by adding 30 mg of SWCNTs soot to 30 mL of 10 g/L (1% w/v) DOC and tip sonicated for 45 min in an ice bath, then centrifuged at 16 650 × g for 1 h. Next, 1.2 mL of SWCNTs solution was mixed with 0.4 mL (Au@Pt/SWCNTs 1:3) or 2.0 mL (Au@Pt/SWCNTs 5:3) of Au@Pt colloidal solution. The mixtures were placed on a rotary shaker at 200 rpm for 3 h. Then they were diluted with ultrapure water, and vacuum-filtered to obtain SWCNTs or Au@Pt/SWCNTs films. The films were repeatedly washed on the filter with ultrapure water until no visible gray coloration from the SWCNTs remained. Subsequently, the films were rinsed with isopropanol to remove residual impurities. The thorough washing ensured all of the surfactant from SWCNTs and Au@Pt solutions was eliminated, as verified by the lack of detectable Br in EDS analysis. We previously showed that the presence of surfactants had a significant impact on the conductivity and toxicity of the prepared surfaces.^9,11^ PET substrates were cleaned with ethanol and ultrasonication (10 min) and dried on a hot plate (80° C). Filtered films were transferred onto the preheated PET using ethanol and gentle pressure, then cut into approximately 25 mm^2^ pieces and mounted on copper holders using double-sided adhesive tape. Electrical contact was established with silver paste and reinforced with copper tape. Finally, electrodes were masked with Teflon tape containing a 3 mm-diameter hole to define a sensing area for the working electrode.

### Electrochemistry

#### Basic Electrochemical Measurements

Cyclic voltammetry (CV) characterized the electrochemical behavior of the SWCNTs, Au@Pt/SWCNTs 1:3, and Au@Pt/SWCNTs 5:3 electrodes. We analyzed three electrochemical systems: an outer-sphere redox probe (OSR): Ru­(NH_3_)_6_^3+/2+^, a surface-sensitive redox probe: ferricyanide/ferrocyanide Fe­(CN)_6_^3‑/4–^, and an element-sensitive probe, H_2_SO_4_.

The 1 mM Ru­(NH_3_)_6_^3+/2+^ solution was prepared in 1 M KCl and scanned within a potential range of −500 to +200 mV at a scan rate of 50 mV s^–1^. CVs of 2 mM Fe­(CN)_6_^3‑/4–^ in 0.1 M KCl were obtained over a range of −100 to +500 mV, at 50 mV s^–1^. H_2_SO_4_ (2 mM in ultrapure water) was degassed with N_2_ for 15 min before measurement and scanned between −200 to 1400 mV, scan rate 50 mV s^–1^.

#### DA Measurements

A 10 mM DA stock solution was prepared in a N_2_-purged PBS, and continuously purged to minimize oxidation/polymerization during storage. PBS used for electrochemical testing was not degassed to mimic physiological conditions. Electrodes were stabilized with 2–3 scans in PBS, after which DA was spiked, stirred for 20 s, and measured. The DA oxidation peak potential was determined for each electrode by recording CVs of DA in PBS at 50 mV s^–1^ between −100 to +350 mV. Peak current and scan rate relationship was studied under identical potential limits at 5–200 mV s^–1^. CV peaks of 1–200 μM DA were recorded to evaluate the linearity and sensitivity of the electrodes. Biofouling resistance was tested by comparing responses in 10 μM DA before and after 30 min incubation in 4% (w/v) BSA in PBS at 38° C. After incubation, the electrodes were gently rinsed with ultrapure water to remove loosely bound BSA.

#### H_2_O_2_ Measurements

A 10 mM H_2_O_2_ stock solution was prepared in PBS, and stored at 8° C to prevent thermal decomposition. The oxidation peak was determined from CVs of H_2_O_2_, after 2–3 blanks scans in PBS at 50 mV s^–1^. The potential range was 100–700 and 100–500 mV for Au@Pt/SWCNTs 1:3 and Au@Pt/SWCNTs 5:3, respectively. Chronoamperometry (CA) was employed to assess sensitivity and linearity vs 0.5–200 μM H_2_O_2_ in PBS. The step potential was set to the upper limit of the respective oxidation peak identified by CVs to ensure complete oxidation. The initial holding potential was 0 mV for 5 s, followed by 60 s at −600 mV (Au@Pt/SWCNTs 1:3) or −500 mV (Au@Pt/SWCNTs 5:3). Biofouling resistance was evaluated by recording responses in 50 μM H_2_O_2_ before and after 30 min incubation in 4% (w/v) BSA in PBS at 38° C. Electrodes were gently rinsed with ultrapure water after incubation to remove excess BSA.

### Data Analysis

Unless otherwise stated, data are presented as mean ± standard deviation (SD). Statistical analysis was performed using one-way analysis of variance (ANOVA). Differences were considered statistically significant at *p* < 0.05. Unless otherwise stated, all reported differences between the presented data were statistically significant according to the ANOVA test. The number of independent measurements or experimental replicates is indicated for each experiment:

AFM images (2.5 μm × 2.5 μm, 512 × 512 pixels) were analyzed using the MountainsSPIP software (ver. 9.3.10663, DigitalSurf, France). Topography images were flattened using a third-order line-by-line leveling before ISO 25178 roughness values were calculated (*N* ≥ 3). Here, N is the number of independent AFM regions analyzed per sample. For tube-width distribution analysis, particle detection was performed on the flattened AFM topography images using an 11 × 11 smoothing filter and a height-pruning threshold of 3% of the height span, S_*z*_. The resulting particle-size data were used to construct the apparent lateral aggregate-size distributions.

For c-AFM current maps, the current display cutoffs were set either in the 0–1 nA or 0–25 nA range, N = 3.

The ECAs were used to calculate the surface energy using the Oss-Chaudhury-Good method, with surface tension values of the probe liquids taken from the same source.^20^ *N* ≥ 3 drops per surface. Data were analyzed in Attention Theta software (Version 4.1.0).

For 4-point probe measurements, 101 readings were collected from N = 5 locations to minimize bias.

XPS values were acquired N = 5 per platform.

Electrochemical data (*N* ≥ 3) were analyzed using Gamry Echem Analyst and Python; statistical analysis and data visualization were performed in Python. For CA analysis, independent current–time peaks were collected for each electrode condition in PBS and after sequential addition of each analyte concentration. For each condition, the replicated values were first averaged, including the PBS measurement, which was treated as the baseline peak. The current response for each concentration was then calculated by first averaging the current and then extracting the values within the 5.49–5.50 s analysis window. Background correction was performed by subtracting the averaged PBS current value from the averaged current values obtained at each analyte concentration within the same analysis window. Therefore, the reported analyte response represents the change in current relative to the corresponding PBS baseline for that electrode condition. For BSA experiments, the same replicate-averaging procedure was applied. However, here, the same electrode was first characterized in PBS and analyte solution before BSA incubation. After BSA incubation, the same electrode was measured, first in PBS to establish the BSA-treated baseline and then after analyte addition. The post-BSA analyte responses were corrected relative to the corresponding post-BSA PBS baseline, allowing the effect of BSA incubation on both the baseline current and analyte response to be evaluated. Finally, pre- and post-BSA baseline-corrected currents were compared by quantifying the percentage change in ΔI values following BSA incubation.

The limit of detection (LOD) was calculated using the following eq 1:

1 $$X_{\mathrm{LOD}}=3.3\frac{\sigma_{\mathrm{Blank}}}{S}$$

where σ_Blank_ is the SD of blank measurements, and S is the sensitivity calculated from calibration curve slopes.

## Results and Discussion

### Material Characterization

#### Surface Morphology

The SWCNTs exhibit a homogeneous and continuous network, where individual nanotubes overlap and entangle (Figure 1a). In some regions, the CNTs form thicker aggregates. However, less often so, there are strand-like structures present. Despite these local variations, the overall morphology remains uniform, suggesting that the surface acts as an integrated cohesive network.

**Figure 1:**
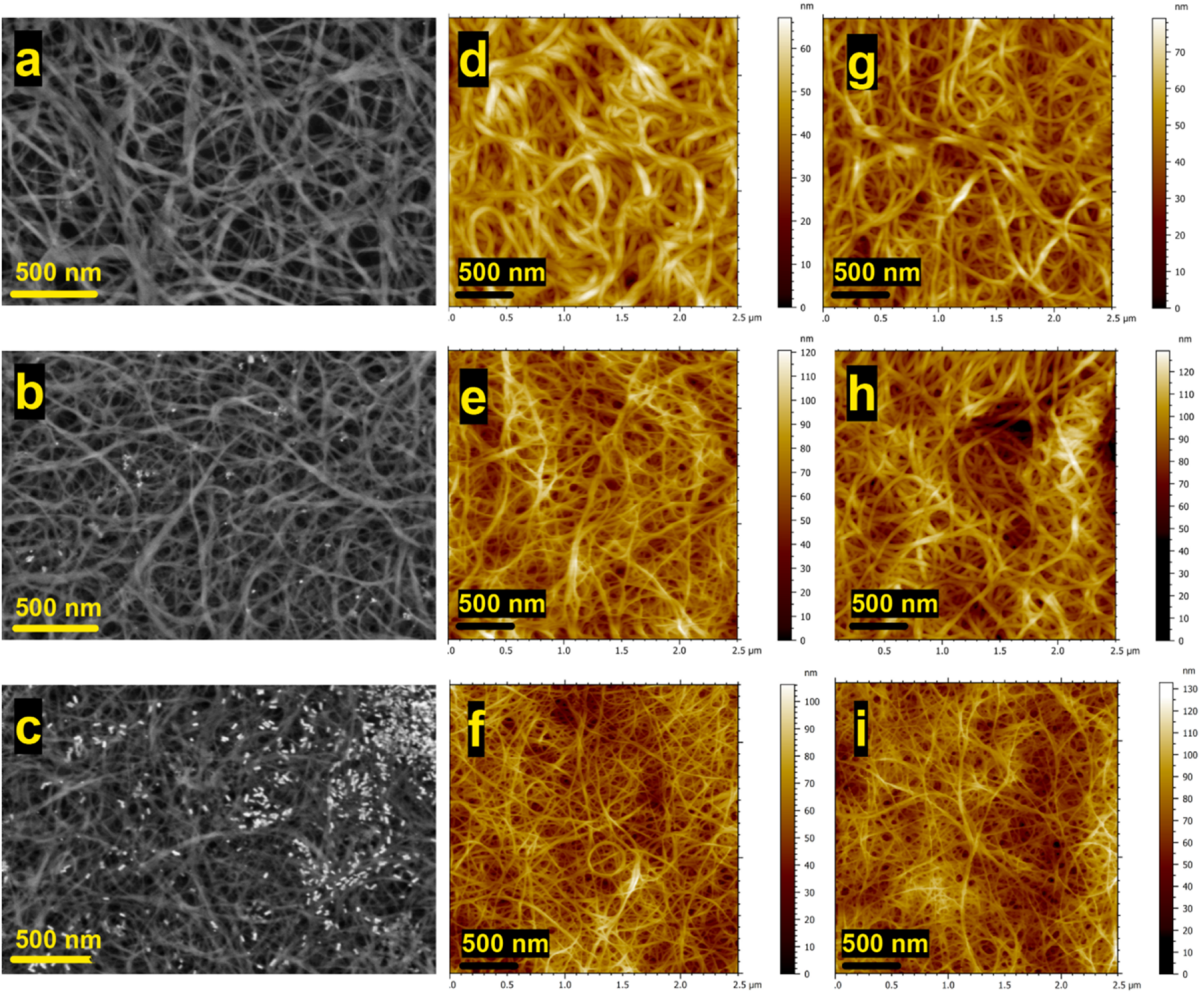
Scanning electron microscopy images (backscattered electron micrographs) of a) pristine SWCNTs, b) Au@Pt/SWCNTs 1:3, and c) Au@Pt/SWCNTs 5:3; atomic force microscopy images of before and after BSA incubation of d, g) pristine SWCNTs, e, h) Au@Pt/SWCNTs 1:3, and f, i) Au@Pt/SWCNTs 5:3, respectively.

For Au@Pt/SWCNTs 1:3, Au@Pt NRs visible in SEM images are scarcely distributed along the nanotube surfaces (Figure 1b). However, their incorporation does not cause a major alteration in the overall morphology compared to pristine SWCNTs. The aggregates appear slightly less compact, and the cavities are somewhat smaller. This modest change in structure may be attributed to the presence of surfactant molecules or metallic NRs in the Au@Pt solution, which likely reduced the tube–tube interactions during the mixing process, due to the interactions between their electromagnetic forces and changes in zeta-potential.

In contrast, the Au@Pt/SWCNTs 5:3 sample displays a drastically different surface morphology (Figure 1c). The amount of Au@Pt NRs is substantially higher, and their dispersion is less uniform, with regions where the particles merge and form film-like layers over the SWCNTs framework. The EDS elemental maps show these film-like layers in a much clearer way (Figure S1). The cavities are further reduced in size, and much fewer thick carbon aggregates can be observed.

AFM imaging supports the same overall trend, with a significant progressive decrease in the thickness of the SWCNTs aggregates as the metallic particle loading increases (Figures 1, S2). The corresponding tube-width distributions (Figure S2) show a shift toward smaller aggregate sizes with increasing Au@Pt NR content.

Additionally, based on the AFM values, the Developed Interfacial Area Ratio (S_dr_) (Table S1), which describes the percentage increase in the true surface area relative to the projected area, rises to approximately 3-fold and 5-fold that of pristine SWCNTs for Au@Pt/SWCNTs 1:3 and Au@Pt/SWCNTs 5:3, respectively. This growth indicates a progressively more textured surface with a larger accessible area.

Point Density (S_pd_) describes the number of surface peaks per unit area, where higher values indicate denser nanoscale surface features. Compared with pristine SWCNTs, S_pd_ increases by approximately 1.2-fold and 2.7-fold for Au@Pt/SWCNTs 1:3 and Au@Pt/SWCNTs 5:3, respectively, indicating a clear increase in surface feature density with higher metal loading.

Comparison of the Autocorrelation Length (S_al_) values reveals a decrease moving from pristine SWCNTs toward Au@Pt/SWCNTs 5:3. Because S_al_ captures horizontal variations in the surface, this reduction indicates a shorter distance (e.g., tube thickness) over which one can move sideways before the surface morphology changes, confirming the thinning of the SWCNTs aggregates; however, the difference is not statistically significant.

Overall, the SEM images together with the AFM parameters (Table S1) show that Au@Pt NRs progressively reorganize the SWCNTs bundles into a more open and finely structured network. The increase in S_dr_ and S_pd_, along with the decrease in S_al_ at higher metal loadings, points to the formation of many small surface features on a less dense and aligned nanotube network, which most likely leads to a more porous, exposed carbon surface with a higher presence of edge sites and defects.

CNTs aggregation is driven by strong intertube van der Waals interactions and is particularly pronounced in SWCNTs.^21^ A variety of physical methods, including ball milling, ultrasonication, and thermal processing, have been employed to disrupt CNT bundles while largely preserving the intrinsic sp^2^ carbon framework; however, these approaches do not affect all bundles uniformly and can induce tube shortening, fragmentation, or broad length distributions due to mechanical stress or thermal effects. In parallel, chemical strategies such as covalent functionalization, polymer-assisted dispersion, biomolecule incorporation (e.g., DNA), and deposition-based approaches including electrodeposition, plating, and spray-based techniques have been widely explored; however, these methods either alter the nanotube lattice or substantially modify the surface chemistry through composite formation or complete coverage with secondary materials, often rendering it chemically distinct from pristine CNTs.^16,17^ To the best of our knowledge, there are no reports demonstrating nanoparticle-induced debundling of SWCNTs followed by the formation of a solid film suitable for chemical sensing applications, even in cases where SWCNTs are decorated with metallic nanoparticles in solution via surface functionalization. Here, we show that incorporation of metallic nanoparticles enables physical rearrangement and separation of SWCNTs while retaining the native nanotube framework, resulting in films composed of the same carbon building blocks but with a reorganized assembly.

#### Surface Energy Properties

Understanding how surface energy governs wettability is essential to interpret interfacial properties and predict performance. In practice, static contact angles of several probe liquids with known surface tension components (γ^DISP^, γ^+^, γ^–^, respectively γ^tot^) are used to decompose the solid surface energy into dispersive and polar (acid–base) contributions. Here, we compared pristine SWCNTs with Au@Pt-decorated SWCNTs at two loadings (Au@Pt/SWCNTs 1:3 and 5:3) using ultrapure water, EG, and DIM as probe liquids (Figure 2).

When probed with water (γ^DISP^ = 21.8 mN·m^–1^, γ^+^ = 25.5 mN·m^–1^, γ^–^= 25.5 mN·m^–1^, γ^tot^ = 72.8 mN·m^–1^), pristine SWCNTs exhibit a high contact angle of 113.2° ± 1.0°, confirming a strongly hydrophobic surface (θ more than 90°). Incorporation of Au@Pt NRs leads to a subtle decrease in water contact angle to 106.7° ± 3.9° for Au@Pt/SWCNTs 1:3, and a pronounced drop to 55.4° ± 1.9° for Au@Pt/SWCNTs 5:3. This indicates that at low metal loading the surface remains hydrophobic, whereas at high loading the surface becomes hydrophilic (θ less than 90°), consistent with a substantial change in surface chemistry and/or roughness.^22^ The significant decrease in water ECAs upon increasing the Au@Pt content is in line with the higher affinity of metallic nanostructures toward polar and electrostatically driven interactions compared to the largely nonpolar carbon surface,^23–25^

A similar trend is observed for EG (γ^DISP^= 29.0 mN·m^–1^, γ^+^= 1.92 mN·m^–1^, γ^–^ = 47.0 mN·m^–1^, γ^tot^ = 48.0 mN·m^–1^), which is a predominantly polar-negative (electron-donor) liquid. The contact angle decreases from 54.9° ± 3.4° on SWCNTs to 50.4° ± 2.4° on Au@Pt/SWCNTs 1:3, and further to 32.4° ± 0.2° on Au@Pt/SWCNTs 5:3, roughly half of the value on pristine SWCNTs. Increasing the metal content strongly enhances the wettability of the surface toward polar liquids.

In contrast, DIM (γ^DISP^ = 50.8 mN·m^–1^, γ^+^= 0, γ^–^ = 0, γ^tot^ = 50.8 mN·m^–1^), which is purely dispersive (polar neutral), exhibits only minor changes (not statistically significant) across the series: 20.9^°^ ± 0.9° for SWCNTs, 22.1° ± 1.9° for Au@Pt/SWCNTs 1:3, and 19.6° ± 0.8° for Au@Pt/SWCNTs 5:3. The consistently low DIM contact angles indicate that the dispersive component of the surface energy is high and remains similar for all three platforms. This is likely due to the strong presence of SWCNTs, their mostly nonpolar nature, and the fact that the net mass of SWCNTs remains constant across all three platforms.

Taken together, the strong decrease in contact angles for water and EG, but the almost unchanged values for DIM, indicate that the principal effect of Au@Pt NRs decoration is to modify the polar (acid–base) contribution of the surface rather than the dispersive one. This is consistent with the surface-energy decomposition (results not shown): the dispersive term is the dominant contribution, while the polar part is governed mainly by the polar-negative (γ^–^) component, with the polar-positive (γ^+^) component being essentially negligible. Liquids such as DIM, which have γ^+^ ≈ γ^–^ ≈ 0, primarily follow dispersive interactions with the solid, whereas polar liquids such as water and EG, with dominant negative/and or positive γs in comparison to their γ^DISP^s, are sensitive to specific acid–base interactions and hydrogen bonding. The fact that only the polar probe liquids reflect the effect of Au@Pt loading, therefore, suggests that the metal NRs introduce or expose additional polar sites and alter the balance between hydrophobic and hydrophilic interactions at the composite surface.

#### Surface Conductivity

Sheet resistance and resistivity increase with a higher loading of Au@Pt NRs on the sensing platform (Table S2). The samples contain SWCNTs in roughly a 2:1 semiconducting-to-metallic ratio.^12^ In mixed networks, metallic SWCNTs provide highly conductive paths, while semiconducting ones add resistance. The overall conductivity depends on metallic connectivity, which is limited by their lower abundance (one-third of the total) and easily disrupted bundle structure, reducing uninterrupted low-resistance channels.

The pristine SWCNTs network exhibits approximately one-third the resistance of the Au@Pt/SWCNTs 1:3 composite and one-quarter lower values than the Au@Pt/SWCNTs 5:3 sample, corresponding to 2.8- and 3.8-fold higher conductivity, respectively (Table S2). The observed increase in resistance may be attributed to a few phenomena. First, the incorporation of Au@Pt NRs, with their inherent nanoscale dimensions (Figure S3), can significantly affect the electrical conductivity of the electrode. Metallic nanoparticles and ultrathin metal films generally exhibit lower conductivity than their bulk counterparts because charge transport is strongly influenced by finite size, morphology, and microstructure. Nanoparticle-based or discontinuous ultrathin films’ conductivities rely on tunneling and percolation between separated metallic regions, which is far less efficient than transport through a continuous crystal. Even when a continuous film is formed, enhanced surface, interface, and grain-boundary scattering, along with small and misoriented grains and rough interfaces, drastically shorten the electron mean free path and increase resistivity.^26,27^ Second, the altered state of the SWCNTs can also help explain the decrease in conductivity. The NR-SWCNTs mix may disturb the tube–tube connections in the SWCNTs network due to the present charges, and the better dispersion of the nanotubes caused by introducing a larger volume of surfactant solution could further reduce the number of conductive pathways, leading to a lower overall conductivity. In short, the disruptive changes to network morphology appear to dominate any charge-transfer benefits that Au or Pt might offer. In the pristine films, SWCNTs assemble into thick, well-oriented bundles that create extended pathways with relatively few junctions, enabling efficient charge transport. Once metallic NRs are introduced, these bundles break into numerous thinner segments, producing many more tube–tube intersections (Figure 1). The resulting increase in junctions increases scattering and, consequently, the overall resistance. This behavior aligns with prior studies, showing that dense, aligned SWCNT bundles consistently conduct better than more fragmented or disordered networks.^12,28–31^

Consistent with the results above, c-AFM measurements (Figure 3) provide spatially resolved evidence that increasing Au@Pt NR loading progressively changes the intrinsic charge-transport behavior of the SWCNTs networks. The c-AFM current maps shown here were acquired at a single positive bias (100 mV); however, measurements at zero, negative, and positive bias values (results not shown) reveal a consistent response. Under zero bias, no measurable current was detected, indicating the absence of spontaneous charge transport in an unbiased state. Upon application of either negative or positive bias, the same localized regions reproducibly exhibit enhanced conductivity, independent of the polarity of the applied voltage. The lack of bias-selective or threshold-like behavior rules out semiconducting-dominated transport and indicates conduction through metallic SWCNTs. This aligns with the mixed SWCNTs composition, where the metallic fraction forms highly conductive pathways.

**Figure 2:**
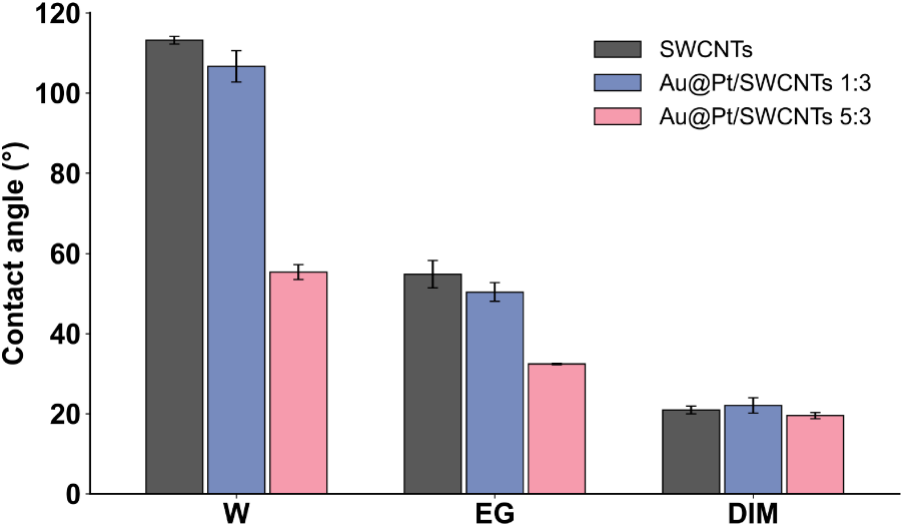
Equilibrium contact angles of black) pristine SWCNTs, blue) Au@Pt/SWCNTs 1:3, and pink) Au@Pt/SWCNTs 5:3 for water, ethylene glycol (EG), and diiodomethane (DIM).

For the case-by-case investigation, by applying two different current thresholds to the c-AFM maps, the same platforms can be interpreted from two complementary perspectives. In the first perspective, only the highly conductive regions are considered, using a threshold of ≥25 nA (Figure 3a-c). In these maps, regions with current ≥25 nA are shown as the more conductive domains (pink), while regions below this threshold are shown as less conductive areas (green). From this viewpoint, increasing Au@Pt NRs modification leads to a progressive decrease in the number, size, and connectivity of highly conductive domains. The SWCNTs platform shows more continuous high-current pathways (nearly 10%), whereas the modified, especially the Au@Pt/SWCNTs 5:3 platforms, show increasingly isolated conductive regions (dropping to nearly 5%) (data not shown). Correspondingly, the lower-conductivity regions expand and become nearly uninterrupted, indicating disruption and fragmentation of the most conductive SWCNTs transport pathways.

**Figure 3:**
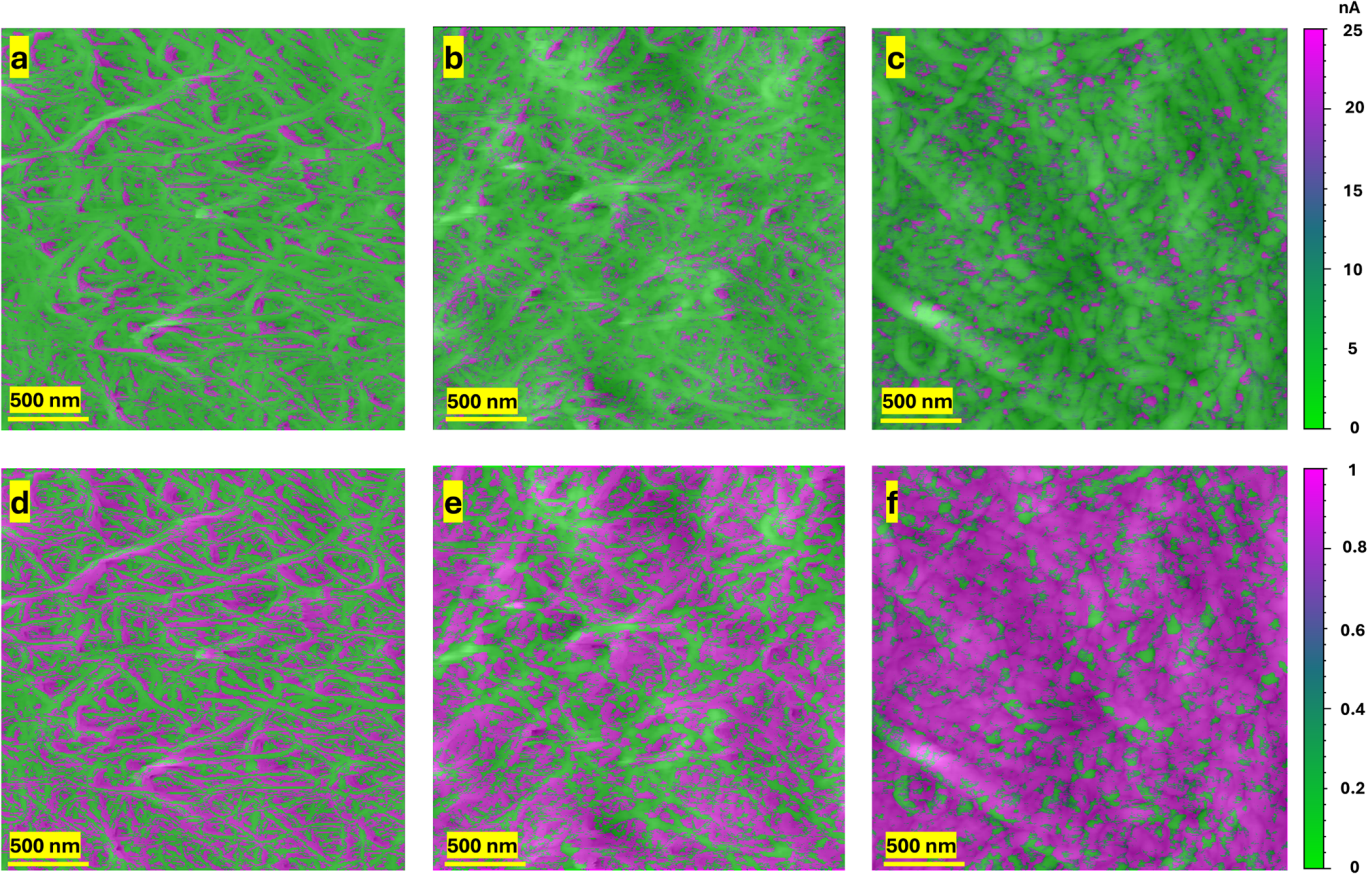
Conductive atomic force microscopy images with overlaid height and current maps of a, d) pristine SWCNTs, b, e) Au@Pt/SWCNTs 1:3, and c, f) Au@Pt/SWCNTs 5:3, for cutoff values of 25 nA (top row) and 1 nA (bottom row). The bias potential was 100 mV for all images.

In the second perspective, the maps are evaluated using a lower current threshold of ≥1 nA (Figure 3d-f), which reflects whether the surface exhibits at least a minimal level of local conductivity. Here, regions with current ≥1 nA are shown as conductive areas (pink), while regions below this threshold are shown as lower-conductivity areas (green). Under this criterion, the opposite trend becomes apparent: the conductive area increases with increasing Au@Pt NR loading, from approximately 35% for pristine SWCNTs to approximately 66% for Au@Pt/SWCNTs 5:3 (Table S3). This suggests that gradual incorporation of Au@Pt NRs distributes low-level conductive regions over a larger fraction of the surface, producing a more broadly conductive and electrochemically accessible platform. Therefore, while Au@Pt NR decoration disrupts the highly conductive, long-range SWCNTs pathways observed at ≥25 nA, it simultaneously increases the spatial coverage of locally conductive regions detectable at ≥1 nA. These observations support a dual effect of NR modification: fragmentation of the strongest current transport channels, together with expansion of the overall electrochemically accessible conductive surface.

This change can be attributed to the debundling of SWCNTs into thinner strands, which increases the number of nanotube–nanotube junctions and disproportionately affects the already limited metallic conduction network. As a result, the already restricted charge transport becomes confined to fewer, spatially isolated regions where favorable junction configurations persist.

Another viewpoint on the cause of change in conductivity can be that in pristine films, the presence of thick SWCNTs bundles ensures that partial wrapping or masking by semiconducting SWCNTs does not fully disrupt metallic conduction pathways and allows extended conductive regions to remain interconnected. With increasing modification and debundling, thinner SWCNTs strands become more easily surrounded by semiconducting SWCNTs, masking the contribution of metallic pathways. This reduces electrical coupling between the c-AFM probe and the underlying conductive regions, leading to enhanced spatial isolation and more dominance of nonconductive areas as the degree of modification increases.

### Electrochemical Studies

#### Basic Electrochemical Characterizations

Several experiments were conducted to understand the interfaces better (Figure 4). Although graphitic surfaces such as SWCNTs are often described as electrically neutral and inert, in aqueous dispersions they typically exhibit a slightly negative surface potential due to oxygen-containing defects or adsorbed charged molecules of the surfactant.^32–36^ Modification of the electrode surface with the metallic NRs altered the Ru­(NH_3_)_6_^3+/2+^ electron-transfer response compared to the pristine SWCNT electrode (Figure 4a). Specifically, the anodic peak currents increased by 23% and 50% for the Au@Pt/SWCNTs 1:3 and Au@Pt/SWCNTs 5:3 electrodes, respectively (see Table 1). To assess whether the observed differences in peak currents originate from changes in electron-transfer kinetics, the peak-to-peak separation (ΔE_p_) was examined. ΔE_p_ remains significantly larger than the theoretical reversible value of 57 mV for all electrodes, which can be expected from nanostructured films with finite conductivity. Also, the variations among the electrodes are minimal and non statistically significant. This indicates that the reaction kinetics are not substantially affected by surface modification (approximately the same I_Pa_/I_*Pc*_ values) and therefore cannot account for the differences in peak current and voltammetric response. Consequently, the enhanced peak currents should be attributed to other factors, primarily related to the intrinsic characteristics of the redox probe. Due to its positive charge and nonadsorptive behavior, Ru­(NH_3_)_6_^3+/2+^ interacts preferentially with the exposed, slightly negatively charged, debundled carbon on the modified electrodes. This electrostatic interaction increases the effective interfacial area, reflected by the increased current density (*j*), normalized to the electrode’s geometric surface area (changing from 0.68 ± 0.01 for pristine SWCNTs to 0.83 ± 0.06 and 1.00 ± 0.06 for Au@Pt/SWCNTs 1:3 and Au@Pt/SWCNTs 5:3, respectively).

**Figure 4:**
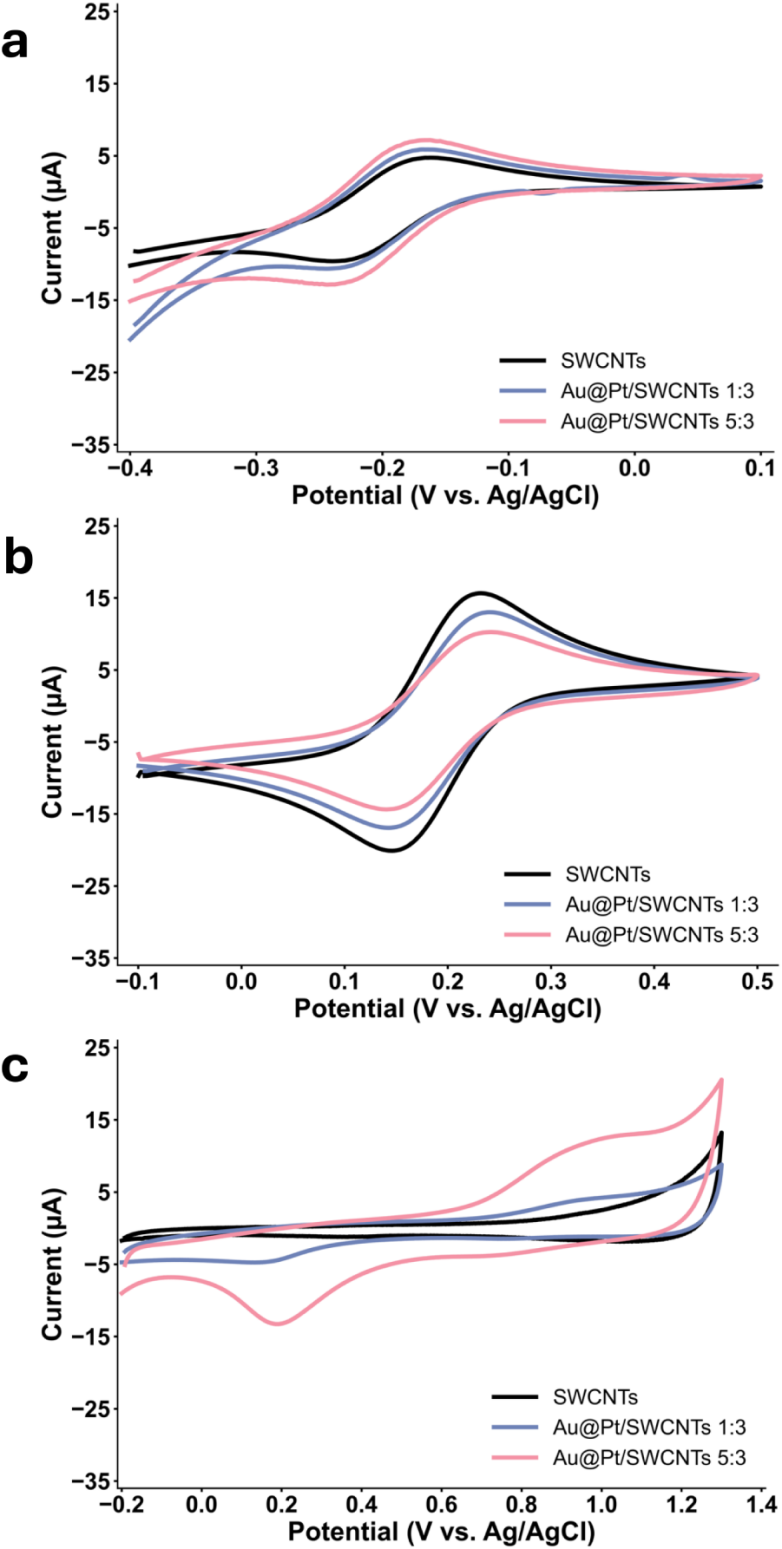
Cyclic voltammograms of pristine SWCNTs (black), Au@Pt/SWCNTs 1:3 (blue), and Au@Pt/SWCNTs 5:3 (pink) in a) 1 mM Ru­(NH_3_)_6_^3+/2+^, b) 2 mM Fe­(CN)_6_^3‑/4–^/0.1 M KCl, and c) H_2_SO_4_ 2 mM, scanned at 50 mV s^–1^.

**Table 1:** Results of the CV Measurements vs. 1 mM Ru­(NH_3_)_6_^3+/2+^/1 M KCl and 2 mM Fe­(CN)_6_^3‑/4–^/0.1 M KCl at 50 mV s^–1^

	I_Pa_ (μA)	I_*Pc*_ (μA)	ΔE_p_ (mV)	I_Pa_/ I_*Pc*_	EASA
Ru(NH_3_)_6_^3+/2+^					
SWCNTs	4.8 ± 0.1	9.6 ± 1.8	75.7 ± 4.0^a^	0.50 ± 0.08^a^	
Au@Pt/SWCNTs 1:3	5.9 ± 0.5	10.7 ± 0.6	82.3 ± 7.0^a^	0.56 ± 0.05^a^	
Au@Pt/SWCNTs 5:3	7.2 ± 0.4	12.7 ± 0.2	76.4 ± 2.3^a^	0.56 ± 0.03^a^	
Fe(CN)_6_^3‑/4–^					
SWCNTs	15.7 ± 0.6	20.2 ± 1.3	85.8 ± 3.5^a^	0.78 ± 0.02	5.1 ± 0.2
Au@Pt/SWCNTs 1:3	13.0 ± 0.3	16.9 ± 0.4	97.8 ± 10.6^a^	0.77 ± 0.01	4.2 ± 0.1
Au@Pt/SWCNTs 5:3	10.4 ± 1.7	14.5 ± 1.8	103 ± 23.3^a^	0.71 ± 0.3	3.4 ± 0.6

a No significant difference.

Figure 4b presents the electrochemical response of the electrodes to the Fe­(CN)_6_^3‑/4–^ redox couple. In contrast to the Ru­(NH_3_)_6_^3+/2+^ probe, the Fe­(CN)_6_^3‑/4–^ redox couple undergoes electron transfer through specific adsorption and direct interaction with surface-active sites. As a result, its voltammetric response is highly sensitive to changes in surface chemistry and the availability of electrochemically active carbon sites. Au@Pt NRs modification of pristine SWCNTs reduced anodic peak currents by approximately 11% (Au@Pt/SWCNTs 1:3) and 32% (Au@Pt/SWCNTs 5:3 electrodes) relative to the pristine SWCNT electrode, indicating suppressed Fe­(CN)_6_^3‑/4–^ electro-oxidation on the modified surfaces.

This change suggests that Au@Pt NRs incorporation alters the electrochemical accessibility of the electrode surface. In the conductive SWCNTs network, this interaction is likely through the high exposure of electroactive carbon sites. Since Fe­(CN)_6_^3‑/4–^ is a surface-sensitive redox probe, the higher peak current indicates more favorable interfacial electron-transfer behavior. The progressive loading of Au@Pt NRs likely masks adsorption-competent sites, such as nanotube ends and defect-rich regions of the SWCNTs network, which are preferentially occupied by metallic nanostructures. As a result, the number of accessible reactive sites decreases despite the presence of additional exposed carbon. Consistent with this interpretation, the ΔE_p_ increases (however, not significantly) with NR loading, indicating increasingly sluggish electron-transfer kinetics as reactive sites become masked. The decrease in anodic peak current and increase in ΔE_p_ support a combined effect of electrostatic repulsion and site blocking. The electrochemically active surface area (EASA), estimated using the Randles-Ševčík equation, decreases with increasing Au@Pt NR content, further confirming that the effective active surface area relevant to Fe­(CN)_6_^3‑/4–^ electrochemistry is reduced due to masking of surface-active carbon sites.^37–40^

Compared with the four-point probe measurements, these results suggest that bulk film conductivity and electrochemical activity reflect distinct aspects of charge transport within the nanostructured network. While increasing Au@Pt NRs modification leads to higher sheet resistance, likely due to disruption of long-range conductive pathways, the concurrent debundling of SWCNTs structures increases the number of electrochemically accessible regions. Consequently, outer-sphere probes such as Ru­(NH_3_)_6_^3+/2+^ may exhibit relatively preserved or slightly enhanced responses due to local interfacial accessibility, whereas the more surface-sensitive Fe­(CN)_6_^3‑/4–^ response more closely reflects the increased resistance of the modified films (Figure S4).

Finally, the Pt oxidation peak at ∼200 mV becomes more pronounced with increasing Au@Pt NR loading (Figure 4c), confirming increased Pt incorporation within the SWCNTs matrix. This indicates greater exposure of Pt sites on the electrode. No peaks are observed for pristine SWCNTs within the same potential range, confirming that the peaks arise from the electrode modifications.

#### DA Electrochemical Characterization and Detection

DA is extremely sensitive to adsorption and interfacial charge-transfer kinetics; defect sites, edge-plane carbons, and oxygen groups affect its current response.^41^ In physiological conditions, DA predominantly exists in its protonated, positively charged form (Figure 5). This cationic nature plays a critical role in determining how DA interacts with CNTs and metallic NRs surfaces.

Therefore, weak electrostatic attraction can exist between positively charged DA species and the mildly negative SWCNT surface, promoting physisorption through π–π stacking and van der Waals interactions. This behavior arises because pristine SWCNTs possess delocalized π-electron systems with relatively fewer active sites for adsorption and electron transfer. Nevertheless, previous studies have shown that even minimal defect densities in CNT electrodes are sufficient to support rapid DA electron transfer, owing to strong electronic coupling between the catechol π-system and exposed edge-plane carbons and tube ends.^42–44^ In contrast, Au@Pt NRs typically carry either a positive or near-neutral surface charge, depending on ligand chemistry and crystallographic orientation, and they have a high affinity to occupy tube ends and edge sites of the SWCNTs. As a result, the NRs induce further debundling of the SWCNT network, exposing additional tube ends and defect sites. While this increases the number of carbon regions accessible to DA, many of these newly exposed sites also serve as anchoring points for the NRs, leading to partial occupation of the SWCNTs’ surface by metal. The electrode, therefore, evolves into a mixed interface composed of exposed carbon domains and metal-decorated regions that compete for DA interaction. This trade-off between increased carbon accessibility and metal coverage governs DA adsorption and electron transfer, resulting in a heterogeneous surface where diffusion-controlled transport and surface-confined charge transfer coexist.^41,42,45^

The modification of SWCNTs with Au@Pt NRs induces systematic changes in both faradaic and nonfaradaic currents during DA oxidation (Figure 6a). The most pronounced thermodynamic effect is a statistically significant positive shift in the anodic peak potential (E_pa_), indicating that DA oxidation requires a higher overpotential as metallic NRs increasingly modify the electrode surface. Similar to the Fe­(CN)_6_^3‑/4–^ redox probe, DA undergoes surface-controlled electron transfer and is therefore sensitive to the availability and nature of surface-active sites. However, unlike Fe­(CN)_6_^3‑/4–^, which is negatively charged, DA is positively charged at physiological pH and experiences favorable electrostatic interactions with the slightly negative carbon surface. Consequently, despite partial masking of defect-rich adsorption sites by Au@Pt NRs, the debundled SWCNTs network remains electrostatically attractive and continues to promote DA accumulation at the interface, leading to an overall increase in oxidation current,^46^ (changing the I_pa_ values from 0.24 ± 0.01, to 0.33 ± 0.05, and 0.49 ± 0.10 μA for pristine SWCNTs, Au@Pt/SWCNTs 1:3, and Au@Pt/SWCNTs 5:3, respectively, vs DA 10 μM (Figure 5a)).

**Figure 5:**
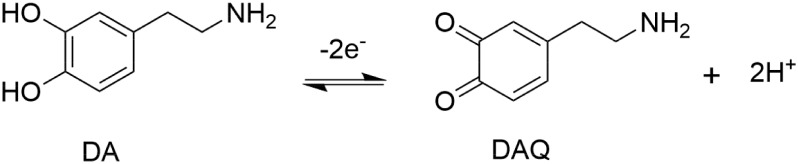
Electrochemical oxidation path of dopamine to dopamine quinone.

At the same time, strong adsorption of both DA and its oxidized form (DAQ) on the metal–carbon hybrid surface stabilizes the oxidized state relative to the reduced one, resulting in an increased peak-to-peak separation (ΔE_p_). This enhanced surface interaction also slows the desorption and diffusion of oxidized species away from the electrode, giving rise to kinetically limited mass transport.^42,45,47,48^ The corresponding increase in the I_pa_/I_pc_ ratio with increasing NR loading is consistent with progressively stronger surface confinement.

In addition, Figure 6a reveals an increase in the background current for Au@Pt/SWCNTs electrodes compared to pristine SWCNTs, reflecting higher double-layer capacitance due to the transition from a compact carbon film to a thinner, high-surface-area SWCNTs network decorated with metallic NRs. Thus, the transition toward a more open, SWCNT-dominated architecture becomes directly apparent in the voltammetric response, complementing microscopic characterization.

The logarithmic dependence of peak current on scan rate provides complementary insight into how the redox process is controlled (Figure 6b and rate-dependent CV peaks in Figure S5). For pristine SWCNTs, the slope of 0.41 in the log­(I_pa_)-log­(ν) plot indicates that DA oxidation is predominantly diffusion-controlled. This is consistent with previous reports showing that relatively smooth graphitic carbon surfaces offer limited adsorption sites, so electron transfer is governed mainly by mass transport in solution.^11,41,49–52^ Upon modification with Au@Pt NRs, the slopes increase to 0.67 for Au@Pt/SWCNTs 1:3 and 0.74 for Au@Pt/SWCNTs 5:3, indicating a shift from mainly diffusion-limited to largely adsorption-controlled kinetics. This behavior is characteristic of heterogeneous metal–carbon hybrid materials, where the mixed metal–carbon interfaces and defects promote stronger DA binding via catechol-metal coordination and π-metal interactions. As these localized surface interactions become dominant, the overall reaction pathway shifts from diffusion in the bulk solution toward surface-confined electron transfer, consistent with an adsorption-dominated regime.^9,42,53^

Figure 6c–e show the behavior of DA on pristine SWCNTs, Au@Pt/SWCNTs 1:3, and Au@Pt/SWCNTs 5:3 across the concentration range of 1–200 μM. The concentration window was chosen to provide a controlled platform in which differences in surface chemistry, adsorption, and electron-transfer kinetics could be quantitatively distinguished. At these micromolar levels, the faradaic response is large enough to show variations in surface reactivity without interference from background noise or mass-transport limitations, enabling a direct comparison between the pristine and modified electrodes.

All three systems exhibited excellent linearity (Figure 6f, Table 2). The progressive increase in slope from pristine → 1:3 → 5:3 indicates that NR coverage promotes DA adsorption by introducing additional heterogeneous surface sites and localized electronic domains. The accompanying heterogeneity is also reflected in the blank signal, since the local dispersion of NRs cannot be reproduced identically and the response varies accordingly, leading to reduced reproducibility and higher %RSD (1:3:3.6%; 5:3:9.6%). This variability elevates the SD of the baseline and, together with the larger background current of the NR-modified electrodes, reduces the peak-to-background contrast. As a result, both factors contribute to the higher calculated LOD for the NR-modified surfaces, while the pristine electrode benefits from a quieter baseline and sharper signal discrimination.

**Figure 6:**
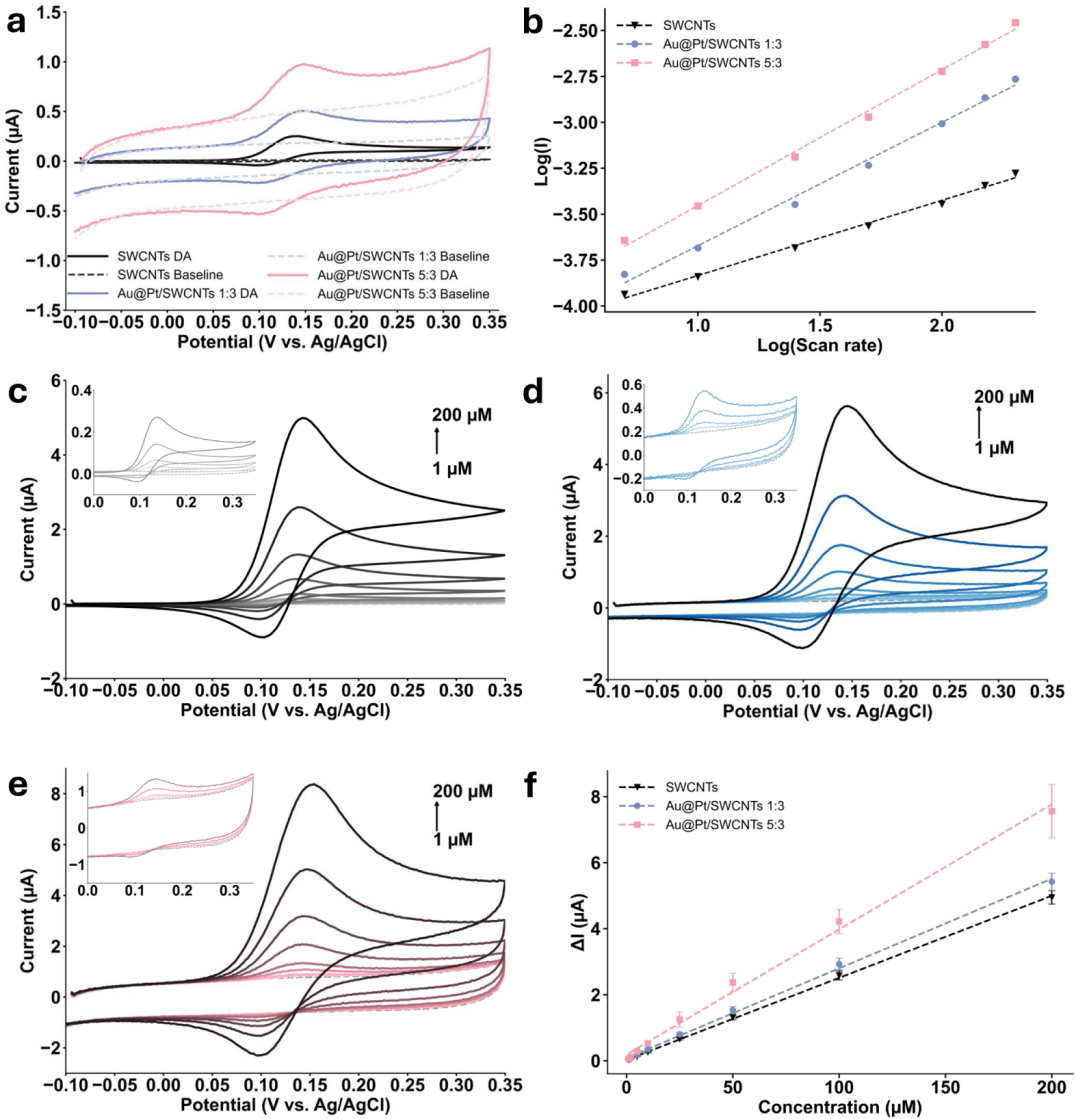
In graphs a–f, black refers to pristine SWCNTs, blue refers to Au@Pt/SWCNTs 1:3, and pink refers to Au@Pt/SWCNTs 5:3, a) Cyclic voltammograms vs PBS and DA 10 μM, b) logarithmic dependence of peak current on scan rate (log­(I_pa_)-log­(ν)), cyclic voltammograms of concentrations series for c) pristine SWCNTs, d) Au@Pt/SWCNTs 1:3, and e) Au@Pt/SWCNTs 5:3 between 1 to 200 μM, inset: zoomed in peaks for background and DA 1–10 μM, f) calibration curves. All scanned at 50 mV s^–1^.

**Table 2:** Key Electrochemical Parameters for Pristine SWCNTs, Au@Pt/SWCNTs 1:3, and Au@Pt/SWCNTs 5:3 toward DA, and for Au@Pt/SWCNTs 1:3 and Au@Pt/SWCNTs 5:3 toward H_2_O_2_; All CVs were Recorded at 50 mV s ^–1^

	Linear range (μM)	LOD (μM)	Sensitivity (μA·μM^–1^·cm^–2^)	ΔE_p_ (mV)	Calibration curve R^2^
DA					
SWCNTs	1–200	0.02	0.352	39 ± 4^a^	0.999
Au@Pt/SWCNTs 1:3	1–200	0.40	0.384	47 ± 4^a^	0.998
Au@Pt/SWCNTs 5:3	1–200	0.62	0.536	51 ± 5^a^	0.995
H_2_O_2_					
Au@Pt/SWCNTs 1:3	1–5	0.127	1.138	425 ± 13	0.999
	5–200				0.998
Au@Pt/SWCNTs 5:3	0.5–2	0.045	7.973	265 ± 7	0.966
	5–200				0.972

a No significant difference.

In this context, the upper end of the tested range (up to 200 μM) allows the system to be probed under higher surface-coverage conditions, making it possible to observe changes in adsorption and possible saturation effects that would not be apparent at lower concentrations. Thus, rather than serving as a low-concentration sensing platform, the calibration data provide mechanistic insight into how metal decoration alters the inner-sphere redox pathway of DA and impacts electron-transfer efficiency.

#### H_2_O_2_ Electrochemical Characterization and Detection

In physiological media, H_2_O_2_ exists predominantly as a neutral molecule, a major difference from DA. Therefore, its oxidation depends on catalytic turnover at Pt sites rather than on adsorption through charge-driven interactions. H_2_O_2_ does not need to bind through specific functional groups or conjugate with the graphitic surface. Instead, its oxidation proceeds at metal sites capable of mediating bond cleavage and intermediate stabilization. Consequently, the Pt sites added onto SWCNTs dictate the reactivity far more than the carbon network itself. However, as the incorporation of Au@Pt NRs leads to the debundling of the carbon network, it effectively increases the available surface area of the carbon scaffold. Because the NRs preferentially anchor at defect sites or at the tube ends, more Pt–C interactions are enabled, and the increased number of exposed and separated tubes provides a greater density of active sites for Pt.

Pt can oxidize H_2_O_2_ either through a direct electron transfer (faradaic) pathway (eq 2) or through a nonfaradaic oxidation pathway (eq 3); The metal enables O–O bond cleavage and the subsequent formation of transient species such as −OOH, −O, or −OH without undergoing a formal change in oxidation state. The balance between these pathways depends strongly on the oxidation state of Pt and the physical profile of exposed sites.^9,54,55^

$$H_{2}O_{2}\to O_{2}+2H^{+}+2e^{-}$$ 2

3 $$2H_{2}O_{2}\to 2H_{2}O+O_{2}$$

Due to the nature of products in the pathways above, the local interfacial pH can decrease during sustained oxidation, which in turn can shift peak potentials at higher concentrations of H_2_O_2_. When Pt is predominantly metallic, as in this study,^9^ H_2_O_2_ can follow a nonfaradaic catalysis, adsorbing, dissociating, and decomposing without changing Pt oxidation state. In this scenario, the metal behaves as a catalytic platform rather than a redox partner, stabilizing intermediates such as −OOH, −O, or −OH through its high density of unoccupied electronic states near the Fermi level.

We^9^ demonstrated that the morphology of these Au@Pt NRs, which is characterized by nanoscale bumps, high-index facets, and a large population of under-coordinated Pt atoms, provides a greater density of such reactive sites. Even subtle changes in surface roughness or facet orientation produced measurable changes in catalytic efficiency. The presence of these particles on the previous carbon platform yielded only diffusion-controlled behavior toward H_2_O_2_, a behavior we hypothesize to be similar on SWCNTs, as H_2_O_2_ does not oxidize like an inner-sphere molecule.

Pristine SWCNTs show almost no response to H_2_O_2_ (Figure S6), confirming that the carbon scaffold is not a catalytic participant. Once decorated with Au@Pt NRs, a clear oxidation signal appears (Figure S7). Notably, the Au@Pt/SWCNTs 5:3 electrode exhibits a significantly more negative oxidation peak position compared to the 1:3 counterpart, indicating lower overpotential for peroxide oxidation and a more catalytically favorable surface. Although the Au@Pt/SWCNTs 5:3 electrode displays a higher background current (consistent with the increased metal loading and the trends discussed previously in the DA and basic electrochemical screening sections) the faradaic response toward H_2_O_2_ is clearly larger.

This difference becomes even more evident in the CA measurements, Figure 7a (baseline-corrected plot shown in Figure S8). When comparing the current response in the absence and presence of 50 μM H_2_O_2_ in PBS, the Au@Pt/SWCNTs 5:3 electrode shows an increased signal compared to the 1:3 platform. After baseline correction (peak current minus background), the normalized response of the 5:3 electrode (7.1 ± 0.9 μA) is approximately 99% higher than that of the 1:3 electrode (3.6 ± 0.4 μA). After retaining background contributions from increased metal loading, the 5:3 configuration maintains a significantly stronger catalytic response, consistent with a higher density of accessible Pt sites and more efficient peroxide oxidation. No current variations is observed for SWCNTs in CA. Figures 7b and 7c present the behavior of H_2_O_2_ on the Au@Pt/SWCNTs 1:3 and 5:3 electrodes in their respective concentration ranges. The baseline-corrected peaks can be seen in Figure S9. On both platforms, the signals of increasing concentrations are distinguishable even in 0.5 s after the potential step, showing highly favorable temporal resolution. For the 1:3 modified surface, the calibration curves (Figure 7d and Table 2) exhibited two distinct linear regions: 1–5 μM and 10–200 μM. The 5:3 platform displayed linearity 0.5–2 μM and 5–200 μM. In both cases, the calibration slopes were noticeably steeper in their lower-concentration windows, reflecting the high catalytic efficiency of the Au@Pt surfaces when peroxide flux is low. The considerably lower LOD in the 5:3 platform demonstrates that the increased metal loading provides a larger population of catalytically active Pt sites (Table 2).

**Figure 7:**
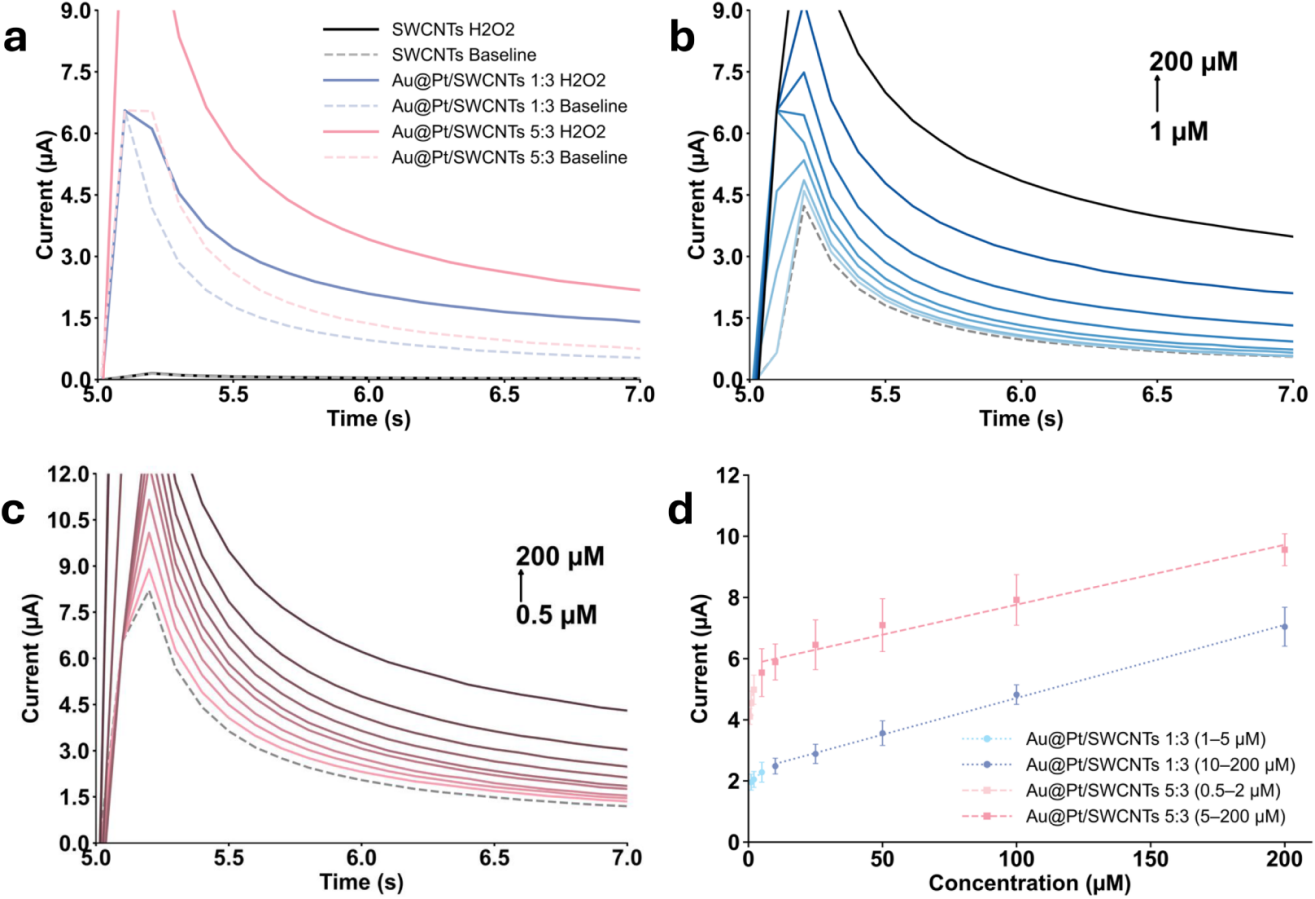
In a–d, black refers to pristine SWCNTs, blue refers to Au@Pt/SWCNTs 1:3, and pink refers to Au@Pt/SWCNTs 5:3, a) Amperometric response vs PBS and H_2_O_2_ 50 μM. Concentration series for b) Au@Pt/SWCNTs 1:3, c) Au@Pt/SWCNTs 5:3 between 1 to 200 μM and 0.5 to 200 μM, respectively. d) Calibration curves according to the amperometric data.

This behavior might be due to the mechanistic picture described earlier: H_2_O_2_ oxidation proceeds primarily at the Pt domains, where the nanostructures provide a high density of accessible, under-coordinated surface atoms that promote chemical reaction for the analyte. As a result, the current response may increase sharply in the low-μM regime, where catalytic sites are far from saturation, and the Au@Pt/SWCNTs 5:3 configuration may benefit from both higher Pt content and greater site accessibility. However, the origin of the biphasic calibration behavior cannot be conclusively assigned based on the present data. Differences in the dispersion and accessibility of Pt sites between sensors may also contribute to the variation in peak reproducibility, as reflected by the %RSD values of 1.2% for Au@Pt/SWCNTs 1:3 and 12.1% for Au@Pt/SWCNTs 5:3. Therefore, the mechanism underlying this response remains unclear and merits further study.

It should be noted that the altered electrochemical response of the Au@Pt NRs/SWCNT films should not be interpreted as a simple additive contribution of two unchanged components. While the catalytic behavior of Au@Pt NRs alone was evaluated in our previous work^9^ and pristine SWCNTs were included here as the carbon-only control, NRs incorporation in the present films also induced substantial reorganization of the SWCNT network. Thus, the electrochemical response reflects the combined influence of Au@Pt catalytic sites and NR-induced changes in SWCNT morphology, surface accessibility, and hydrophilicity.

### Biofouling

In physiological environments, electrochemical sensors are immediately exposed to biomolecules, which limit mass transport, and alter the electrochemical signal. Here, the two target neurotransmitters rely on distinct oxidation pathways at the electrode interface, meaning that protein adsorption may influence their signals unequally. To evaluate this effect, the sensors were incubated in BSA solutions for 30 min, and the electrochemical response before and after incubation was compared. BSA is a soluble protein with a largely polar, charged outer surface and buried hydrophobic regions (including pockets that can accommodate aromatic or nonpolar ligands). Its surface is not uniform: it presents mixed areas in hydrophilic and hydrophobic regions, and these regions can rearrange when BSA contacts a solid interface. Because BSA’s net charge depends strongly on pH (pI ≈ 4.7–5.0), it is typically net negative under neutral aqueous conditions,^56–58^ so interfacial charge distribution becomes a primary determinant of how it approaches and organizes on SWCNTs surfaces. These interactions can influence the local environment encountered by redox-active analytes such as DA, which itself exhibits strong π–π and electrostatic interactions with sp^2^ carbon; similarly, access and reaction pathways for H_2_O_2_, whose electrochemical response is sensitive to local surface chemistry, defect sites, and interfacial organization, may be indirectly modulated by BSA-induced reorganization at the CNTs interface.

AFM images show that BSA binds weakly to thick, bundled, pristine SWCNTs films Figure 1g, which can be consistent with the fact that a bundled network can be very hydrophobic and present a relatively “closed” outer skin: much of the graphitic area is buried inside bundles, and the externally exposed surface can be comparatively low in high-energy anchoring sites, limiting stable attachment even if hydrophobic interactions are available. As the surface becomes more debundled, hydrophilic, and decorated Au@Pt NRs, the accessible surface area increases (more tube ends and junctions are exposed to solution), and the interface becomes richer in nanoscale features that enable binding (cavities and often more edge or defect sites). Also, decorating the debundled network with Au@Pt NRs can amplify this effect by introducing polar metallic domains that act as strong local anchors: negatively charged regions of BSA can be held near neutral-to-positively polarized metal.

All of this allows BSA to contact the surface at many points and wrap or bridge between tubes, which looks macroscopically like encapsulation, clearly observed for Au@Pt/SWCNTs 5:3 (Figure 1i).

XPS measurements were performed to confirm the higher incubation of BSA on the Au@Pt NRs/SWCNTs 5:3 platform compared with pristine SWCNTs. Although the absolute atomic percentages alone may not directly quantify the amount of adsorbed protein, the observed trend in nitrogen content provides evidence of increased BSA presence. Specifically, the N atomic percentage showed a clear increase for Au@Pt NRs/SWCNTs 5:3 compared with pristine SWCNTs (Table S4).

This agrees with earlier studies showing that when BSA forms a monolayer, it spreads differently on hydrophilic and hydrophobic surfaces, with more extensive and uniform coverage on hydrophilic surfaces, effectively blocking a larger fraction of the active sensing area on modified SWCNT platforms.^58,59^

The aim of the following tests was not to validate a long-term antifouling sensor platform, but to examine how nondestructive SWCNT debundling and Au@Pt NR incorporation affect electrode surface properties, electrostatic interactions, and protein adsorption under controlled fouling conditions. Therefore, pristine and modified SWCNT electrodes were exposed to the same protein-containing environment for a defined incubation time to directly compare surface-dependent fouling behavior.

Long-term incubation, repeated fouling-cleaning cycles, and storage stability were not investigated for the present SWCNT/Au@Pt NR system. However, related studies from our group have examined prolonged protein exposure, biofouling, analyte-induced fouling, and fouling-recovery behavior on different electrode platforms.^42,60–63^ In addition, the same Au@Pt NRs showed good reproducibility after approximately three months of storage in our recent H_2_O_2_ sensing study,^9^ with an RSD below 6%, supporting their shelf stability.

#### DA Biofouling

Before incubation, the pristine SWCNTs electrode (Figure 8a) exhibited the expected DA oxidation behavior with a low baseline current, sharp peak definition, and minimal noise. After incubation, the baseline current increased significantly while the DA oxidation and reduction peaks remained clearly visible, and the peak ratio showed little deviation; however, the overall noise level increased, and the DA peak current rose by ∼+25% (however, not significantly). This behavior is consistent with the formation of a hydrated, ion-permeable BSA film that increases the double-layer capacitance without fully blocking charge transfer. Similar trends have been reported for carbon electrodes, which typically resist severe fouling by forming thin, adherent, yet permeable biofouling layers that still allow measurable DA oxidation.^64–66^ However, for nanotubes, because commercially available and research-grade CNTs span a wide range of conductive-to-semiconductive ratios, and because the concentration and nature of interfering species, as well as their growth environments, can vary substantially, direct comparisons across studies remain challenging. In addition, the length and diameter of CNTs (MWCNTs, DWCNTs, and SWCNTs) differ significantly depending on synthesis and processing conditions. Moreover, few studies have examined interactions between BSA and pristine CNTs (particularly SWCNTs) as opposed to CNT-based composites. As a result, it is difficult to establish a universal rule governing SWCNT-BSA interactions, since the intrinsic properties of the fabricated sensory units involved are often not directly comparable.

**Figure 8:**
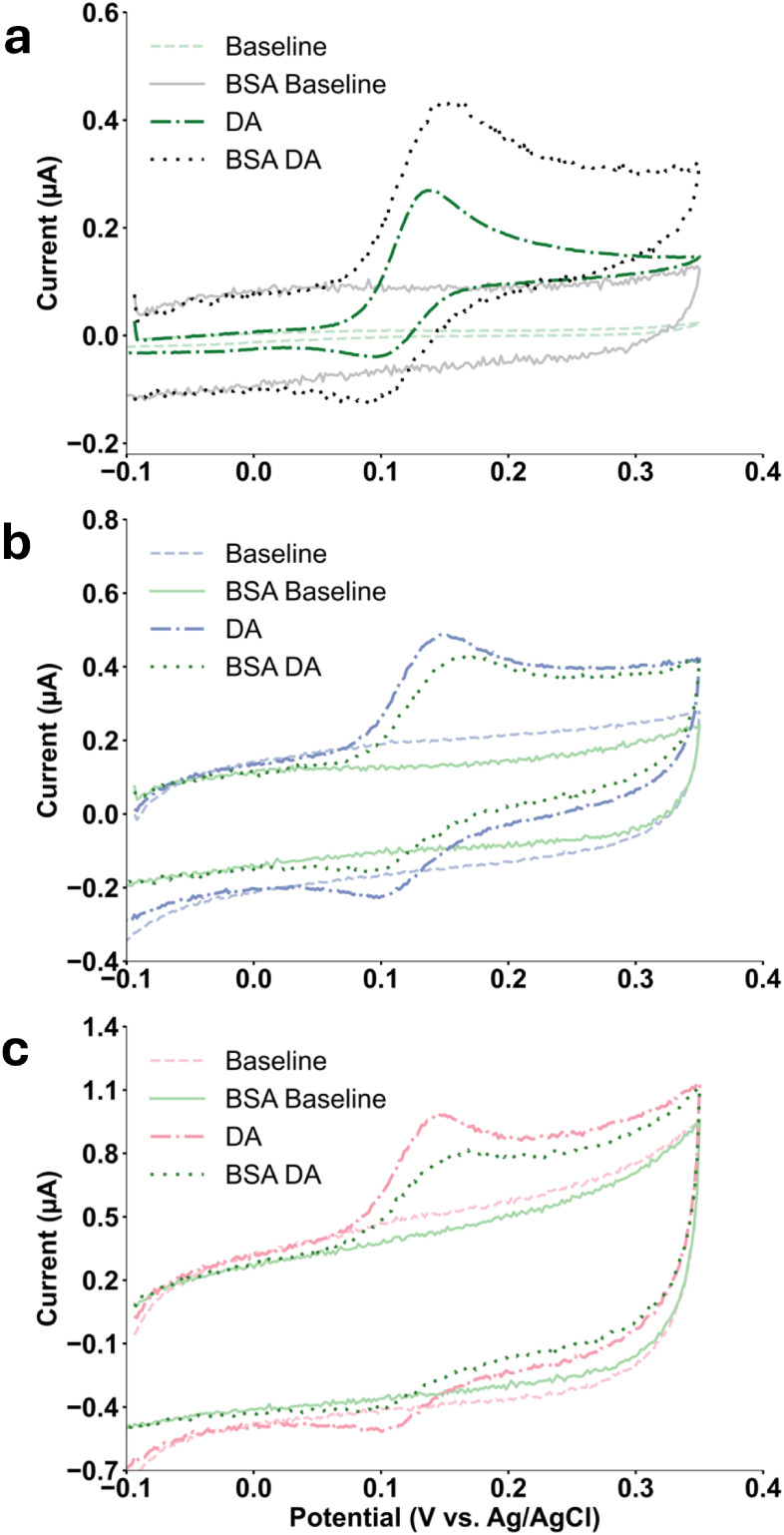
Cyclic voltammograms before and after BSA incubation vs. PBS and DA 10 μM for a) pristine SWCNTs, b) Au@Pt/SWCNTs 1:3, and c) Au@Pt/SWCNTs 5:3, scanned at 50 mV s^−1^.

In contrast, the Au@Pt/SWCNTs 1:3 showed (Figure 8b) a decrease in DA oxidation after incubation, with the peak current decreasing nonsignificantly by ∼−13%, accompanied by a subtle increase in noise but limited change in peak shape or the background current, as if the incubation did not impact the surface chemistry of the sensory unit as much as the pristine SWCNTs. The partial loss of current likely indicates local protein adsorption that restricts surface accessibility, which aligns with earlier observations that metal-containing interfaces develop heterogeneous, resistive domains upon fouling, producing patchy current pathways and elevated noise.^42^

The Au@Pt/SWCNTs 5:3 demonstrated (Figure 8c) the strongest decrease, with the DA peak current reducing significantly by ∼−22%, suggesting that a greater fraction of the electroactive surface was blocked. Because Au and Pt surfaces bind proteins more strongly than carbon, they form denser, more insulating biofilms that suppress DA adsorption and slow electron transfer. However, it seems that the noise did not increase as much as in previous examples, and the background current remained similar to the preincubation state.

Increasing the metal content, therefore, makes the electrodes more susceptible to biofouling, consistent with the progressive loss of current from SWCNTs → Au@Pt/SWCNTs 1:3 → Au@Pt/SWCNTs 5:3 (see Figure S10, Table S5).

Consistently across all electrodes, the peak-to-peak separation for DA increased after incubation, indicating slower electron-transfer kinetics and higher interfacial resistance caused by the BSA layer. This mirrors previous findings that DA-fouled electrodes exhibit broader ΔEp values due to reduced heterogeneous charge-transfer rates at partially blocked surfaces.^42,62,67^ The DA oxidation on these electrodes is largely adsorption-controlled, requiring surface interaction. Protein fouling blocks adsorption sites available for DA, reducing peak currents and broadening peak separation. Carbon-rich electrodes retain detectable DA signals, due to weakly adhered biofouling layers, whereas Au–Pt domains promote stronger chemisorption and greater signal loss.

#### H_2_O_2_ Biofouling

For H_2_O_2_ detection, the pristine SWCNTs electrode showed (Figure 9a) no measurable sensitivity toward H_2_O_2_ before incubation, as no change in the CA peak was observed after addition of the analyte, consistent with previous results. After incubation in BSA, the baseline current of pristine SWCNTs increased dramatically, indicating an elevated background current. Upon H_2_O_2_ addition, the CA peak decreased rather than increased, suggesting it arises not from H_2_O_2_ but from potential-induced partial detachment or restructuring of the adsorbed protein layer. Repeated potential application progressively diminished the peak (data not shown), confirming it is unrelated to H_2_O_2_ oxidation. Although this notable change in background current was observed for pristine SWCNTs following BSA incubation, this does not influence H_2_O_2_ sensing performance, as pristine SWCNTs do not exhibit a measurable electrochemical response toward H_2_O_2_, and therefore no reliable detection limit can be determined.

For Au@Pt/SWCNTs 1:3 (Figure 9b), the background current before and after incubation was nearly unchanged, similar to what was observed for DA, and the CA peak for H_2_O_2_ remained almost identical, resulting in ∼+7% change after background subtraction. This minimal change suggests that biofouling had a limited influence on H_2_O_2_ detection for this electrode. Because the metallic loading in Au@Pt/SWCNTs 1:3 is considerably lower than in Au@Pt/SWCNTs 5:3, adsorbed proteins likely formed a thinner and less continuous film, failing to fully cover or interconnect across the composite network of carbon and metal. As a result, sufficient catalytic sites remained accessible for H_2_O_2_, and the electrocatalytic response was largely preserved.

**Figure 9:**
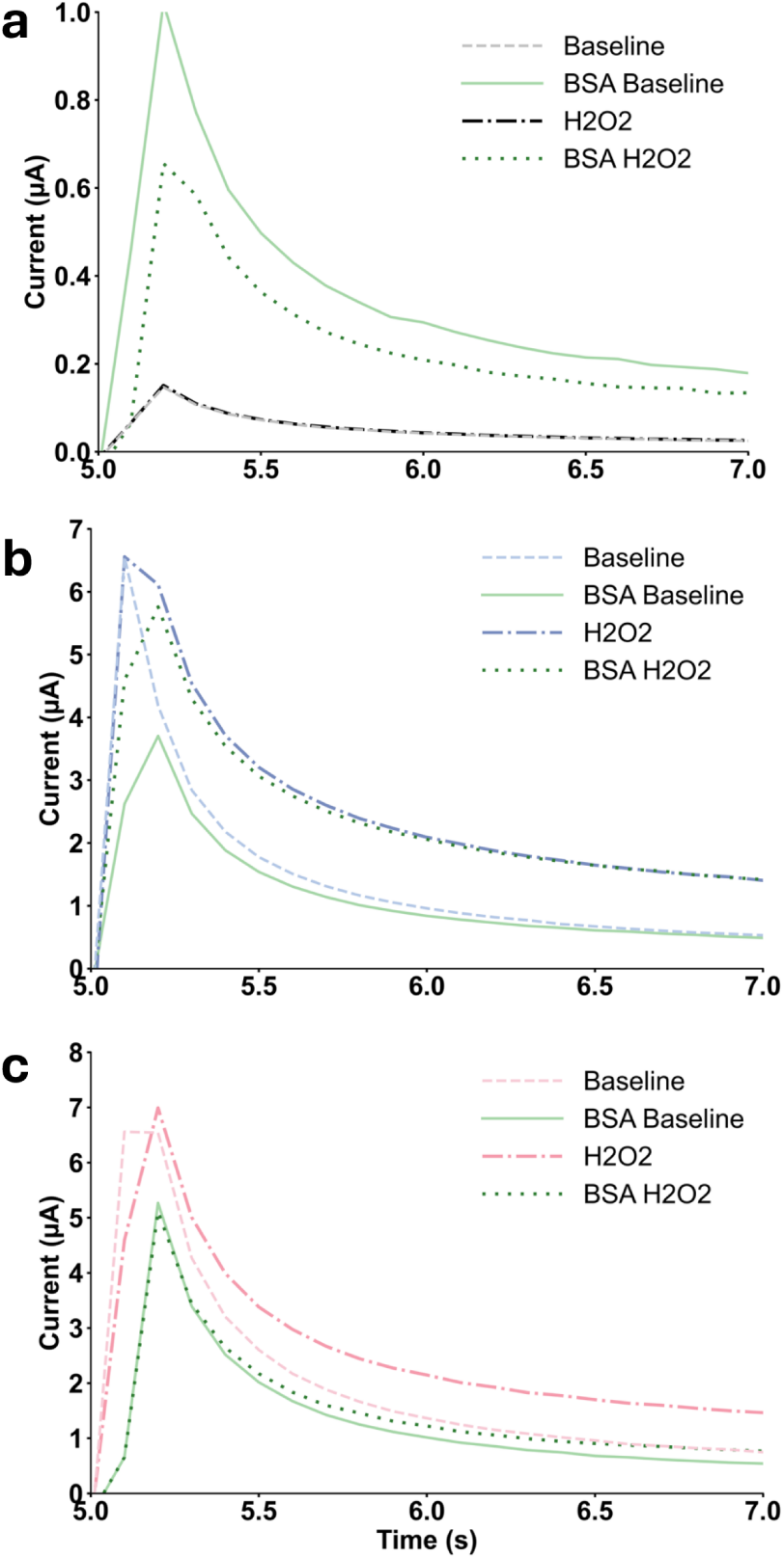
Amperometric response before and after BSA incubation, showing baseline and response to 50 μM H_2_O_2_, for a) pristine SWCNTs, b) Au@Pt/SWCNTs 1:3, and c) Au@Pt/SWCNTs 5:3.

In contrast, Au@Pt/SWCNTs 5:3 exhibited (Figure 9c) substantial biofouling. Following BSA incubation, the background current rose sharply, and after subtraction of the background, the CA peak decreased by ∼−80%, meaning only ∼20% of the original response was retained. This severe suppression may be indicative of a surface that was heavily covered by a protein film, restricting access of H_2_O_2_ to catalytic Pt sites. Since H_2_O_2_ oxidation on Pt relies on direct catalytic decomposition at exposed active sites, dense protein layers reduce the available surface area and impede charge transfer, effectively blocking the electrocatalytic process.^68^ The large drop in current on Au@Pt/SWCNTs 5:3 (see Figure S11 and Table S5), therefore, supports that electrodes with higher metal loading are more susceptible to extensive protein adsorption, which forms thicker and more insulating layers that inhibit catalytic turnover of H_2_O_2_.

## Conclusions

Three SWCNT-based films (pristine SWCNTs, Au@Pt/SWCNTs 1:3, and Au@Pt/SWCNTs 5:3), were fabricated under controlled conditions to systematically investigate how progressive incorporation of Au@Pt NRs influences CNTs organization and electrochemical performance. Increasing NR loading disrupted the strong intertube van der Waals interactions that dominate pristine SWCNT films, leading to progressive debundling and reorganization of the nanotube network. This structural transition resulted in thinner SWCNT strands with enhanced accessible surface area, increased surface feature density, and a reduced horizontal correlation length, reflecting fundamental changes in nanotube aggregation and surface morphology.

NR decoration and debundling increase the hydrophilicity, indicating selective enhancement of the polar and acid–base components of the surface energy. Debundling also increases tube–tube junction density and promotes fragmentation of the metallic conduction network, thereby confining charge transport to fewer favorable pathways.

The electrochemical consequences of these changes strongly depend on the redox mechanism. Interfacial electron-transfer kinetics were unaffected by NRs. However, incorporation of the metallic particles decreased the electrochemical surface area for surface-sensitive probes, consistent with reduced macroscopic conductivity and increased junction resistance.

Electrostatic attraction between DA and progressively debundled SWCNTs drives interfacial accumulation and shifts the response from diffusion- to adsorption-controlled kinetics, highlighting the influence of surface chemistry and nonfaradaic contributions on the measured current.

On pristine SWCNTs, BSA increased background current and noise due to higher double-layer capacitance, while DA oxidation remained largely unaffected. In contrast, increasing NRs loading led to a reduction in electrochemical response without substantial changes in background current or noise, indicating improved suppression of nonspecific electrochemical signals and partial surface stabilization.

In contrast, H_2_O_2_ oxidation is dominated by catalytic mechanisms and is largely insensitive to nonfaradaic contributions. Progressive SWCNTs debundling increased the density of Pt NRs anchored at defects and tube terminals, leading to a higher density of active sites and a strong enhancement of the oxidation current with increasing modification. However, this widespread anchoring rendered the modified platforms more susceptible to biofouling upon BSA incubation. In combination with increased hydrophilicity, a more extensive BSA layer can form over the catalytic sites, thereby amplifying the impact of BSA on the H_2_O_2_ response.

It should be noted that the present work was designed to explore the structure–property relationships and electrochemical mechanisms of the hybrid SWCNT/Au@Pt NRs platforms in simplified model systems. Preliminary BSA studies were used to assess protein-related matrix effects and biofouling tendencies; however, real biological samples such as serum and recovery experiments were not included in the current scope. Future studies will therefore focus on validation in complex biological matrices, including serum recovery experiments, quantitative assessment of matrix effects, and complementary computational modeling to further clarify analyte/surface and protein/surface interactions. Accordingly, the comparison tables (Tables S6 and S7) are included for context rather than direct comparison.

Overall, these results revealed a critical balance between SWCNTs debundling, surface hydrophilicity, and metal loading that governs electrochemical performance in biologically relevant environments. Although increased NRs incorporation enhanced catalytic activity and adsorption-driven processes by introducing additional metal active sites, it also increased hydrophilicity and metal/protein affinity, leading to enhanced biofouling and suppressed effective charge transfer. In contrast, intermediate modification achieved a functional sweet spot, where sufficient debundling and metal dispersion improved electrochemical reactivity without promoting dense, impermeable protein layers. This balance enables sustained sensitivity, reduced nonspecific background, and improved operational stability, underscoring the importance of tailoring hybrid carbon/metal architectures to both the analyte oxidation mechanism and the constraints of complex biological media.

## Supplementary Material



## Data Availability

Data for this article, including raw data, is available on Zenodo (https://zenodo.org/records/21334140).

## Notes

The fabrication steps of Au@Pt NRs, EDS-SEM elemental maps and EDS spectra, AFM values and tube width distributions, mean sheet resistance, resistivity, and conductivity values, TEM images of Au NRs, c-AFM data analysis and their correlation to redox probes, rate-dependent CVs of the platforms vs DA 10 μM, CVs of the platforms vs PBS and H_2_O_2_ 50 μM, background-corrected CAs of the platforms vs H_2_O_2_, XPS atomic percentage analysis, pre- and post-BSA incubation charts and data for DA and H_2_O_2_, and electrode comparison tables for DA and H_2_O_2_.(PDF)
