# Supplementary material for: Controlled Debundling of Single-Walled Carbon Nanotubes (SWCNTs) by Au@Pt Nanorods Enables Mechanism-Dependent Electrochemical Sensing and Biofouling Response
Source: Anal Chem. 2026 Jul 14;98(29):21292–308. doi: 10.1021/acs.analchem.6c00924 (PMC13425561; doi:10.1021/acs.analchem.6c00924)
Supplement: Supplementary file 1 [file ac6c00924_si_001.pdf]

Supporting Information

Controlled Debundling of Single-Walled Carbon Nanotubes (SWCNTs) by Au@Pt Nanorods Enables Mechanism-Dependent Electrochemical Sensing and Biofouling Response

Bahar Mostafiz,[†] Emil Rosqvist,[‡] Ermei Mäkilä,[¶] Vipul Sharma ,[†] and Emilia Peltola*,[†]

† Department of Mechanical and Materials Engineering, University of Turku, Turku, FI-20014, Finland

‡ Laboratory of Molecular Science and Engineering, Åbo Akademi University, Henriksgatan 2, Åbo, FI-20500, Finland

¶ Department of Physics and Astronomy, University of Turku, Turku, FI-20014, Finland

* Corresponding author
Email: emilia.peltola@utu.fi

Table of contents

Nanoparticles fabrication route..S-3

Figure S1. SEM-EDS elemental maps and EDS spectrum....................................S-4

Figure S2. Tube width distribution plot..S-5

Table S1. AFM data..S-6

Table S2. Mean Sheet resistance, resistivity, and conductivity..............................S-6

Figure S3. TEM images of Au@Pt NRs..S-7

Table S3. c-AFM conductive area fraction data..S-8

Figure S4. Conductive areas corrolation with anodic peak currents of OSR and surface sensatives redox probes…………………..………………………………..… S-9

Figure S5. Rate-dependent CV peaks vs. DA S-10

Figure S6. CVs of SWCNTs vs. PBS and H_2O_2...S-11

Figure S7. CVs of modified electrodes vs. PBS and H_2O_2…………….................S-12

Figure S8. Background corrected CAs of modified electrodes vs. H_2O_2 .... S-13

Figure S9. Concentration dependent background corrected CAs of modified electrodes vs. H_2O_2…………..S-14

Table S4. XPS atomic percentage analysis of the incubated platfroms S-15

Figure S10. Pre- and post-BSA incubation current bar chart for DA………….......S-16

Figure S11. Pre- and post-BSA incubation current bar chart for H_2O_2…………....S-17

Table S5. Pre- and post-BSA incubation current comaprison for DA and H_2O_2….S-18

Table S6. Compariosn Table for DA detection S-19

Table S7. Compariosn Table for H_2O_2 detection..S-20

References...S-21

Nanoparticles fabrication route

Au NRs were synthesised in a non-seeding approach. An aqueous CTAB solution (8.33mL, 180 mM) was combined with NaCl (225 μL, 0.1 M), $HAuCl_4.3H_2O$ (180 μL, 0.05 M), and $AgNO_3$ (180 μL, 0.01 M) solutions. This mixture turns to a yellowish-brown hue. After mild-paced shaking to make the whole solution homogenously yellowish-brown, 180 μL AA 0.1M was introduced and the mixture was inverted for 30 seconds to mix, leading to a color shift from yellowish-brown to transparent. Subsequently, ice-cold $NaBH_4$ (10 μL, 3.14 mM) was added, followed by another 30-second inversion, which resulted in a purple solution. The solution was then left undisturbed at ambient temperature for at least 12 hours. Post-synthesis, the samples were transported to a 15 mL falcon tube, placed in 30° C water for the sedimented surfactant to change from crystalline structure to dissolved, and then underwent thrice centrifugation (11000 rpm for 10 minutes each time) and washing with ultrapure water to eliminate surplus reactants and were concentrated by to a final volume of 7 ml.

The Au@Ag NRs were then produced using a PVP-based method to ensure colloidal stability. A mixture of the Au NR solution (0.8 mL) with a PVP solution (4 mL, 1% w/w) was made, followed by the addition of $AgNO_3$ (180 μL, 0.001 M) and AA (100 μL, 0.1 M), with careful stirring after each addition. Then by introducing NaOH (200 μL, 0.1 M), a bluish-green color appeared, indicative of Au@Ag NRs formation.

Au@Pt NRs synthesis were performed by galvanic replacement, when K_2PtCl_4 (250 μL, 0.001 M) was added to the Au@Ag NR solution (5 mL). This solution was stirred magnetically at a pace of 500 rpm and a temperature of 60°C for 5 hours. The formed nanorods were separated by centrifugation at 7000 rpm for 20 minutes and washed multiple times with ultrapure water to purify.

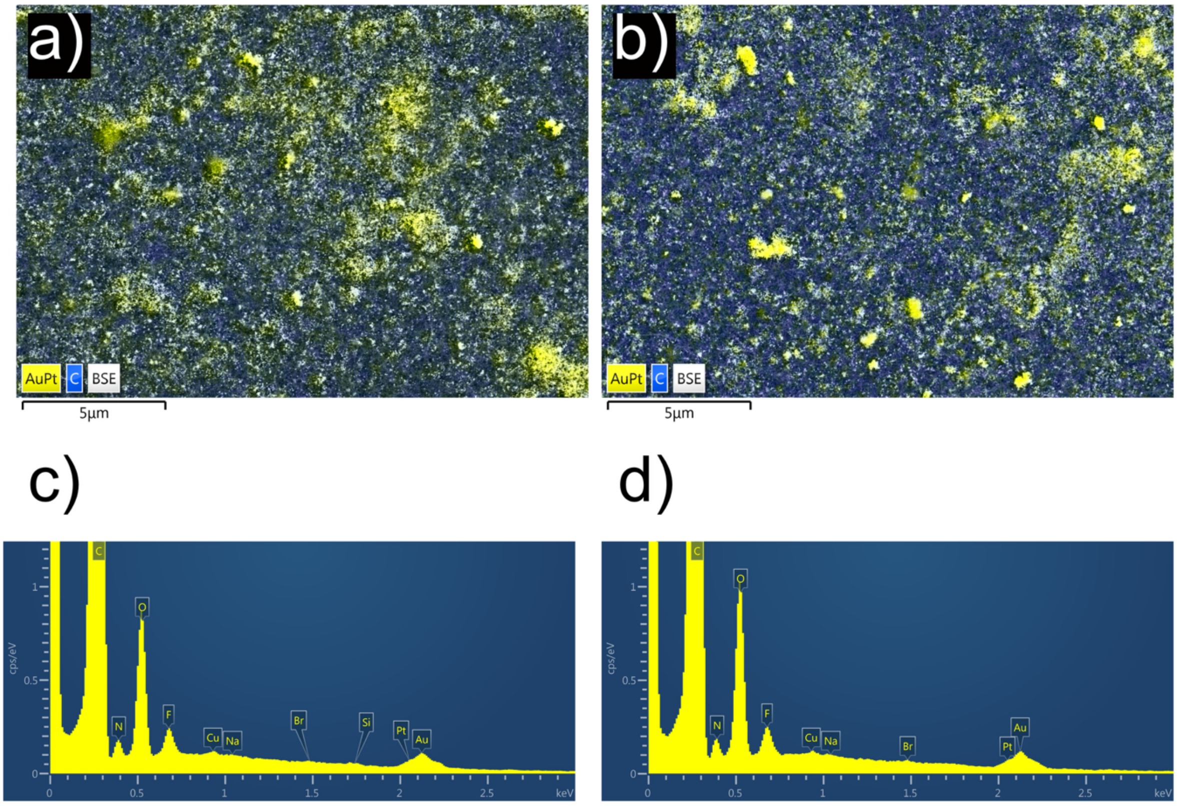

Figure S1. a, b) SEM-EDS elemental maps of the Au@Pt/SWCNTs 5:3 samples. The images were taken from different regions and they show the random distribution of Au@Pt NRs on the SWCNTs structure. c, d) EDS spectrum of the the corresponding samples. As can be seen even for the highest concentration of Au@Pt solution in the Au@Pt/SWCNTs mixture, the Br peak, a prominent element in the structure of CTAB, is below the device detection limit (Br peak location is flagged in the spectra).

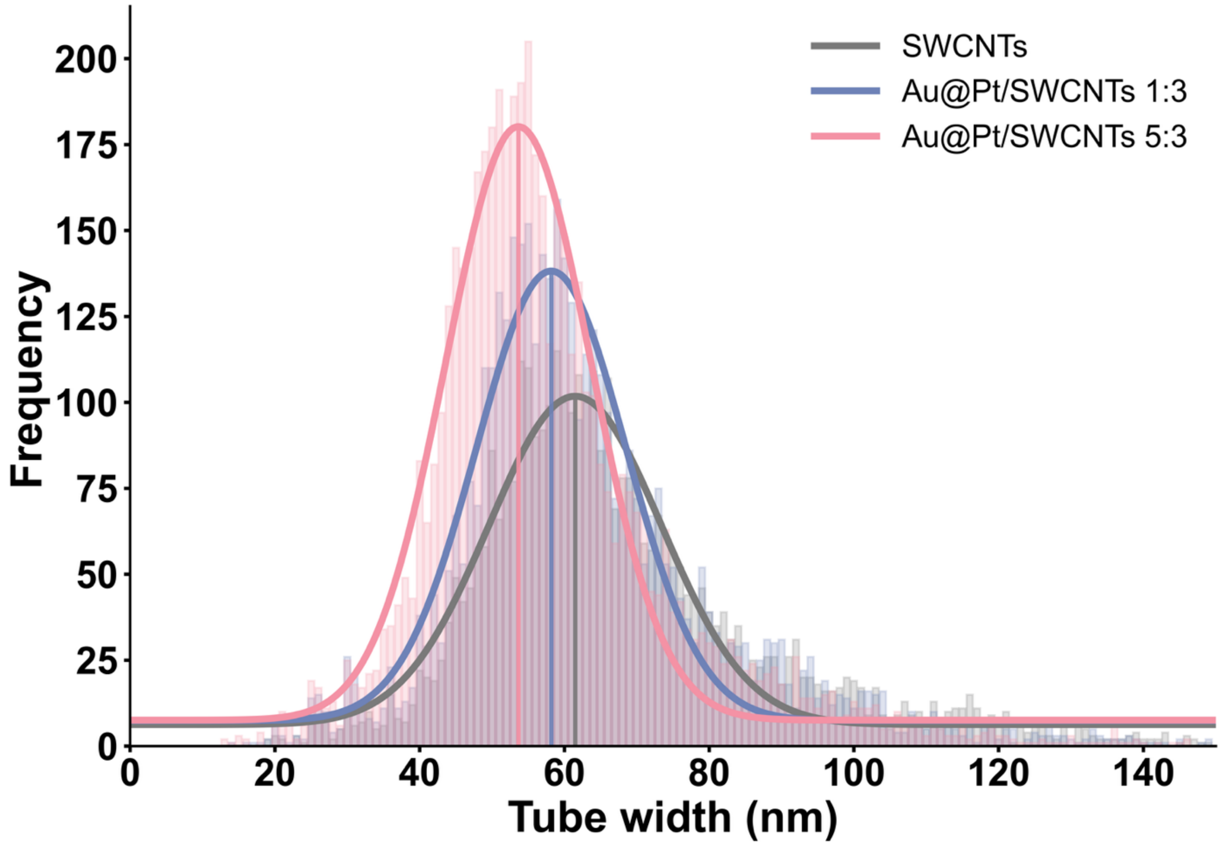

Figure S2. Tube width distribution determined from AFM image analysis for pristine SWCNTs, Au@Pt/SWCNTs 1:3, and Au@Pt/SWCNTs 5:3, shown in black, blue, and pink, respectively. The histograms reveal a gradual decrease in the apparent lateral aggregate size with increasing Au@Pt NR content, supporting progressive thinning and debundling of SWCNT aggregates.

The statistical analysis showed a progressive decrease in the apparent lateral aggregate size upon increasing the Au@Pt NR content. The mean apparent aggregate size decreased significantly from 61 ± 12 nm for the SWCNT sample to 58 ± 10 nm for Au@Pt/SWCNTs 1:3 and further to 54 ± 10 nm for Au@Pt/SWCNTs 5:3. This corresponds to an approximate reduction of 5% and 13%, respectively, relative to the SWCNTs sample. Although these AFM-derived lateral dimensions should not be interpreted as absolute bundle diameters because of tip-convolution effects and the complex morphology of the aggregates, the consistent downward shift in the size distribution supports the proposed trend of progressive SWCNT debundling in the presence of Au@Pt NRs.

Table S1: Obtained AFM data for Pristine SWCNTs, Au@Pt/SWCNTs 1:3, and Au@Pt/SWCNTs 5:3 (N=3).

**Sample**	**S_{dr} (%)**	**S_{pd} (μm^{-2})**	**S_{al} (μm)**
Pristine SWCNTs	6 ± 1	18 ± 4	0.11 ± 0.02
Au@Pt/SWCNTs 1:3	20 ± 7	21 ± 6	0.11 ± 0.02
Au@Pt/SWCNTs 5:3	37 ± 3	48 ± 8	0.06 ± 0.02

Table S2: Mean Sheet resistance, resistivity, and conductivity for pristine SWCNTs, Au@Pt/SWCNTs 1:3, and Au@Pt/SWCNTs 5:3 films (n=5). The standard deviation was calculated using five data points.

**Sample**	**Mean Sheet Resistance** (Ω/sq)	**Mean Resistivity** $(\Omega \text{ x m}) \text{ x } 10^{-6}$	**Mean Conductivity** $(S/\text{m}) \text{ x } 10^{5}$
Pristine SWCNTs	22 ± 1	1.1 ± 0.1	9.0 ± 0.6
Au@Pt/SWCNTs 1:3	63 ± 3	3.1 ± 0.2	3.2 ± 0.2
Au@Pt/SWCNTs 5:3	86 ± 9	4.3 ± 0.5	2.4 ± 0.3

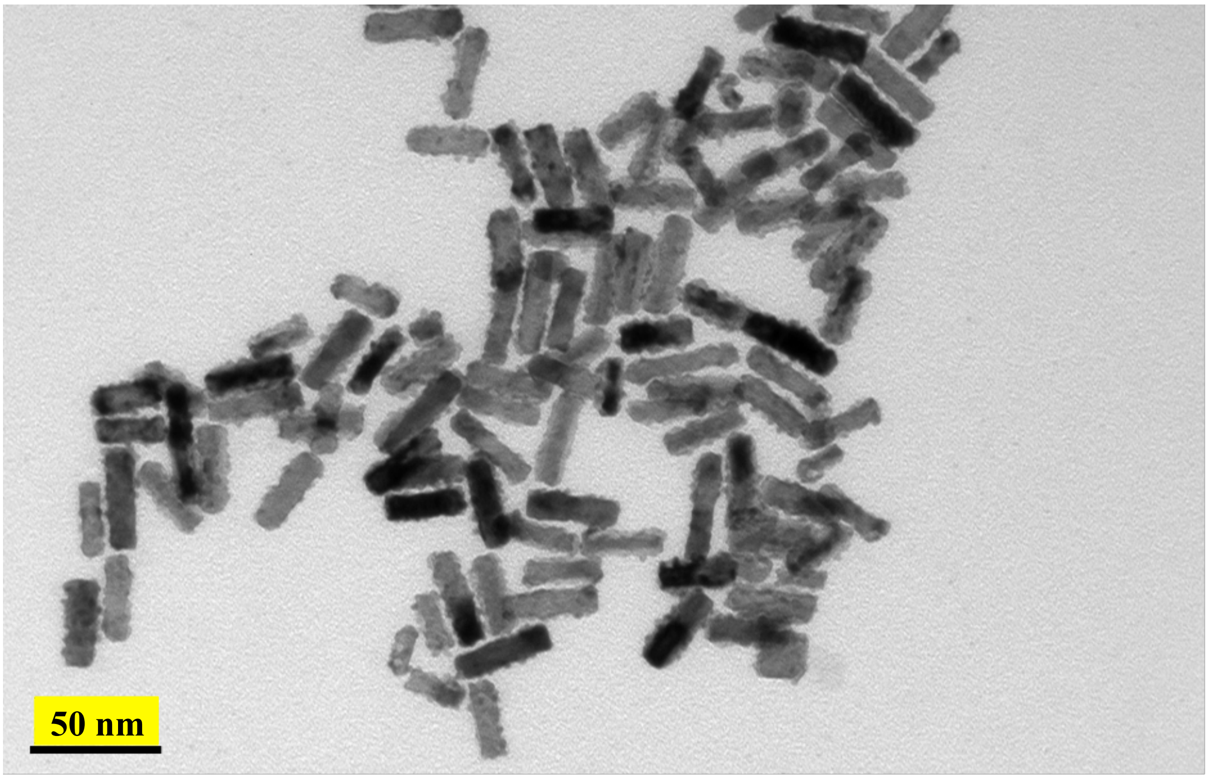

Figure S3. Bright field TEM images of Au@Pt NRs.

Table S3. Fraction of measured points with at least a low (≥1 nA) and high (≥25 nA) surface conductivity on the different samples at a 100 mV bias. (N=3).

Sample	Relative No. of Points w. low surf. conductivity, ≥1 nA (%)	Relative No. of Points w. high surf. conductivity, >25 nA (%)
Pristine SWCNTs	35 ± 3	10 ± 3
Au@Pt/SWCNTs 1:3	45 ± 3	7 ± 2
Au@Pt/SWCNTs 5:3	66 ± 0	5 ± 0

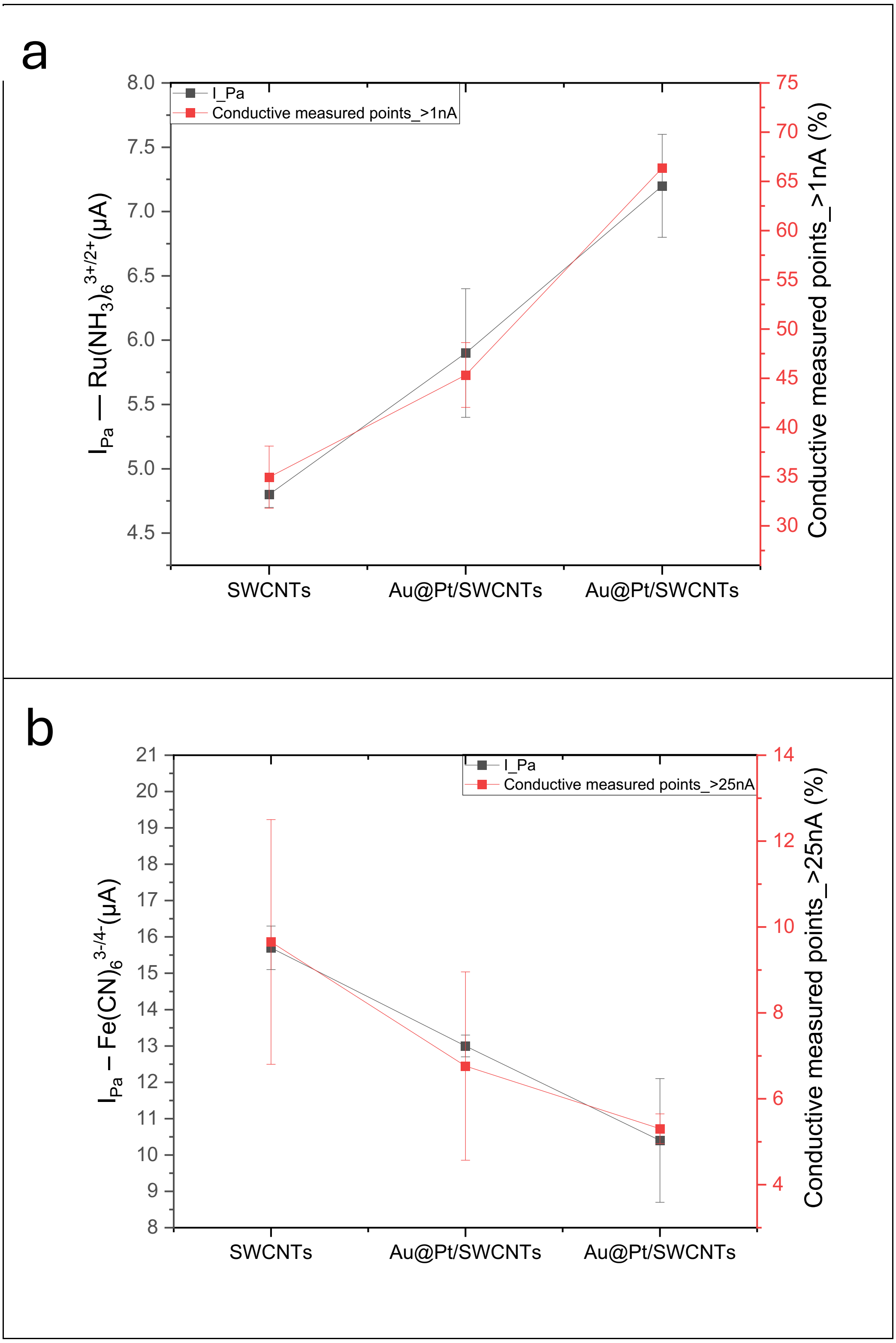

Figure S4. Overlay of a) relative number of measurement points exhibiting *at least* a low surface conductivity (≥1 nA) on anodic peak current value of $Ru(NH_3)_6^{3+/2+,}$ and b) relative number of measurement points with high surface conductivity (≥25 nA) on anodic peak current value of $Fe(CN)_6^{3-/4-}$.

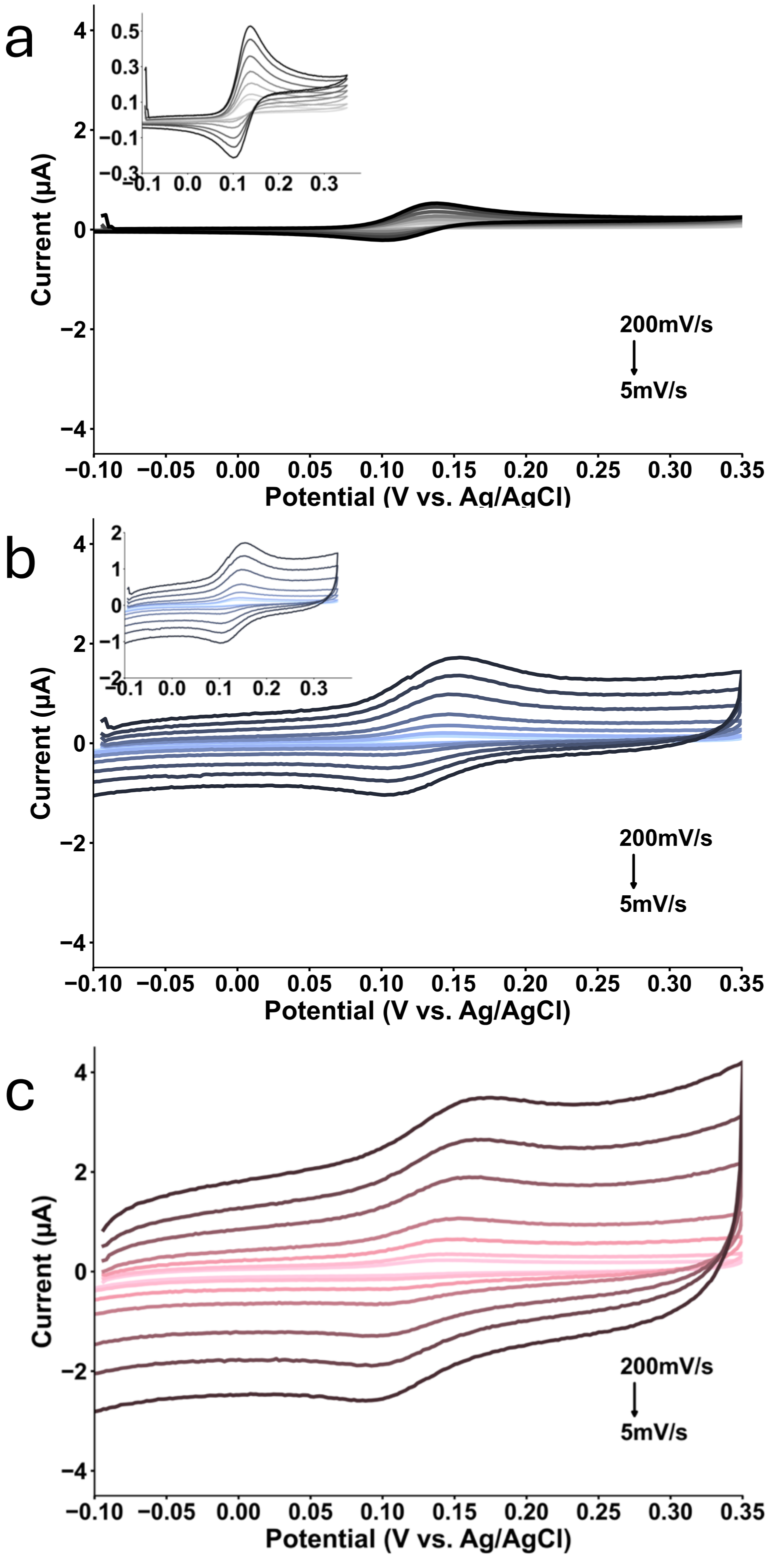

Figure S5. Rate-dependent CV peaks for a) pristine SWCNTs, b) Au@Pt/SWCNTs 1:3, and c) Au@Pt/SWCNTs 5:3 vs. DA 10µM/ PBS. Scan rate: 5 – 200 mV s^{-1}. Insets: zoomed in cyclic voltammograms for better understanding the peak profile.

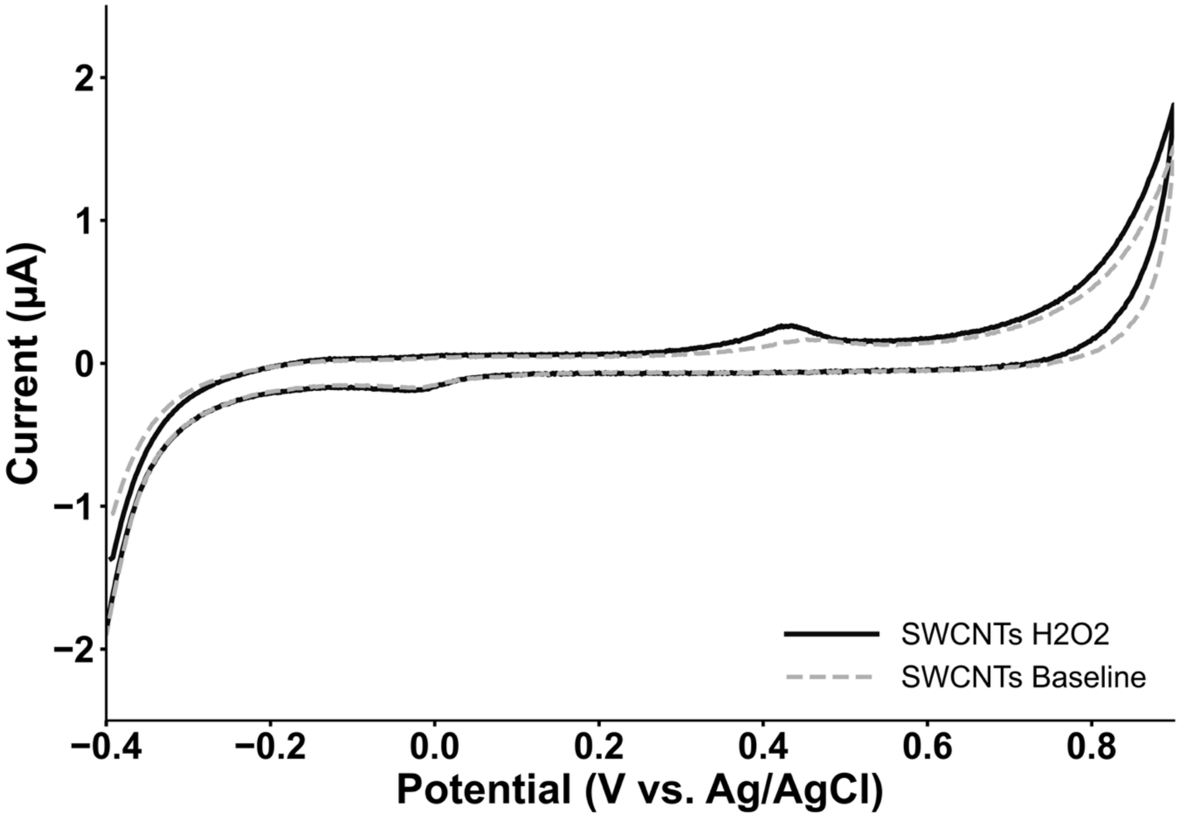

Figure S6. CVs of SWCNTs vs. PBS (dashed line) and H_2O_2 50 µM / PBS (solid line), Scan rate: 50 mV s^{-1}.

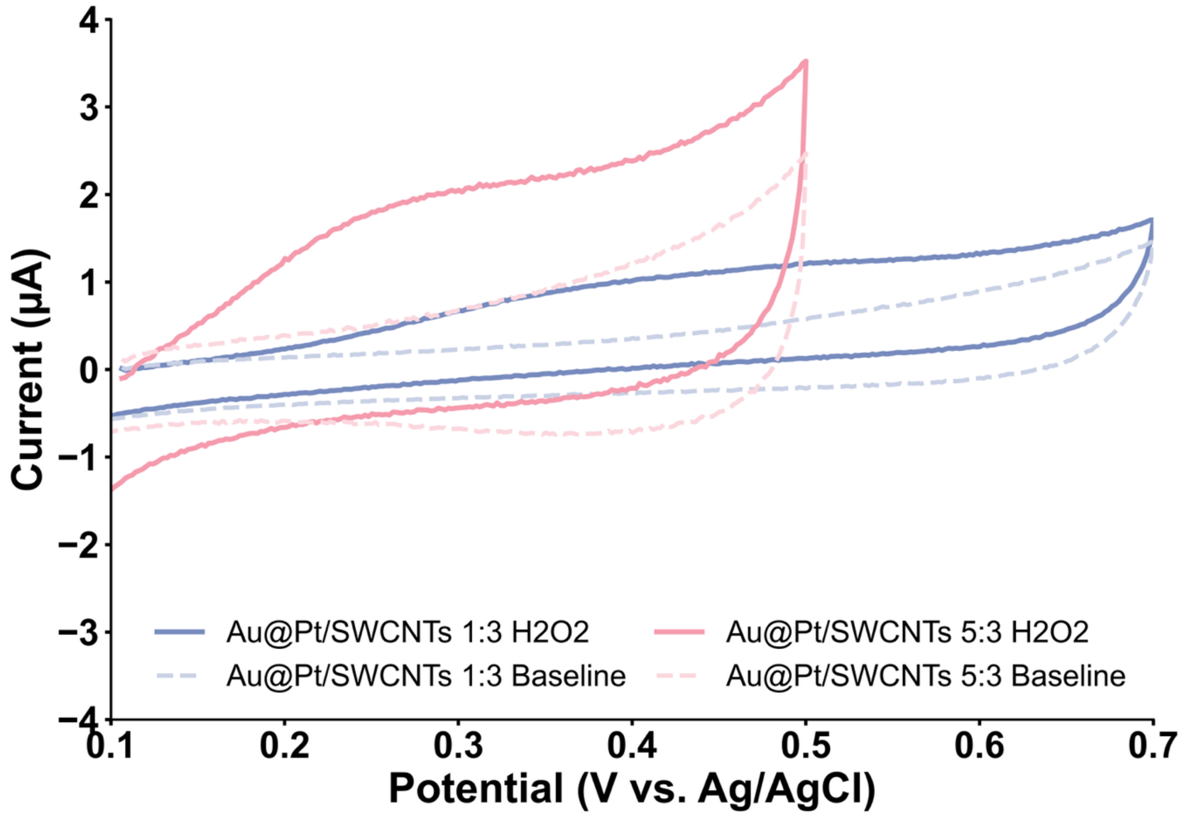

Figure S7. CVs of Au@Pt/SWCNTs 1:3 (blue) and Au@Pt/SWCNTs 5:3 (pink) vs. PBS (dashed line) and H_2O_2 50 µM/ PBS (solid line), Scan rate: 50 mV s^{-1}.

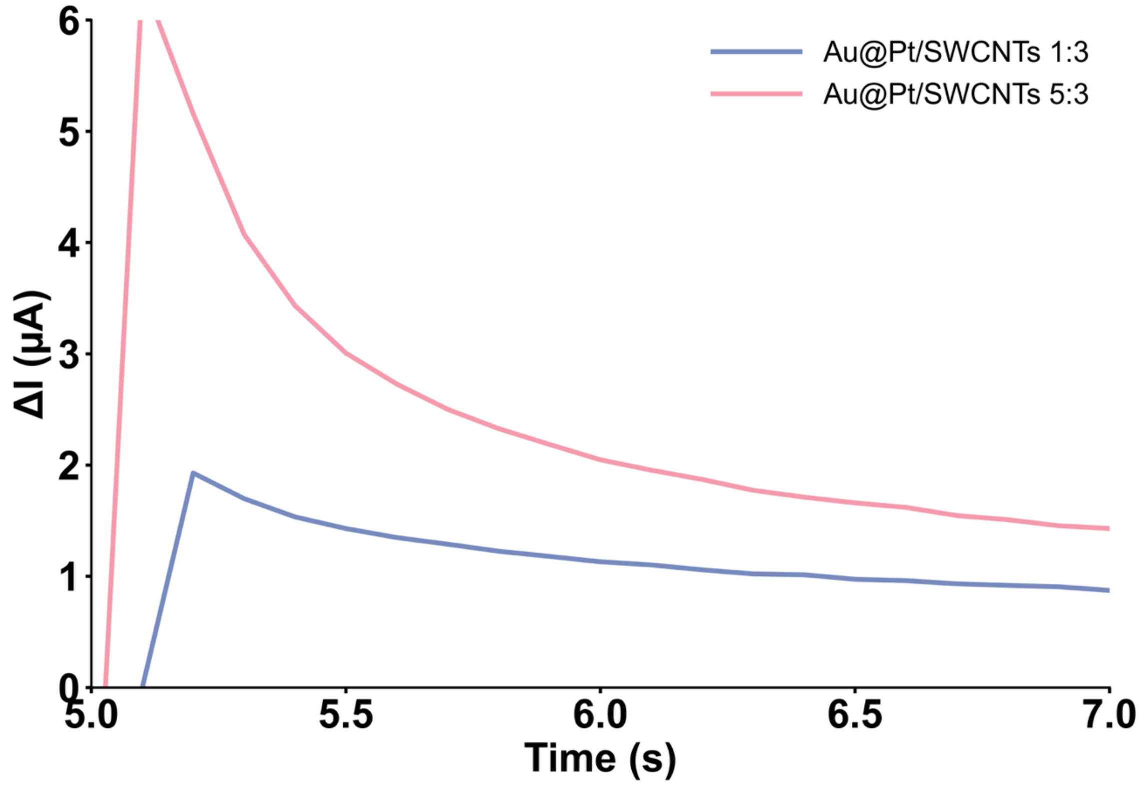

Figure S8. Baseline corrected CAs of Au@Pt/SWCNTs 1:3 (blue) and Au@Pt/SWCNTs 5:3 (pink) vs. H_2O_2 50 µM/ PBS, Scan rate: 50 mV s^{-1}.

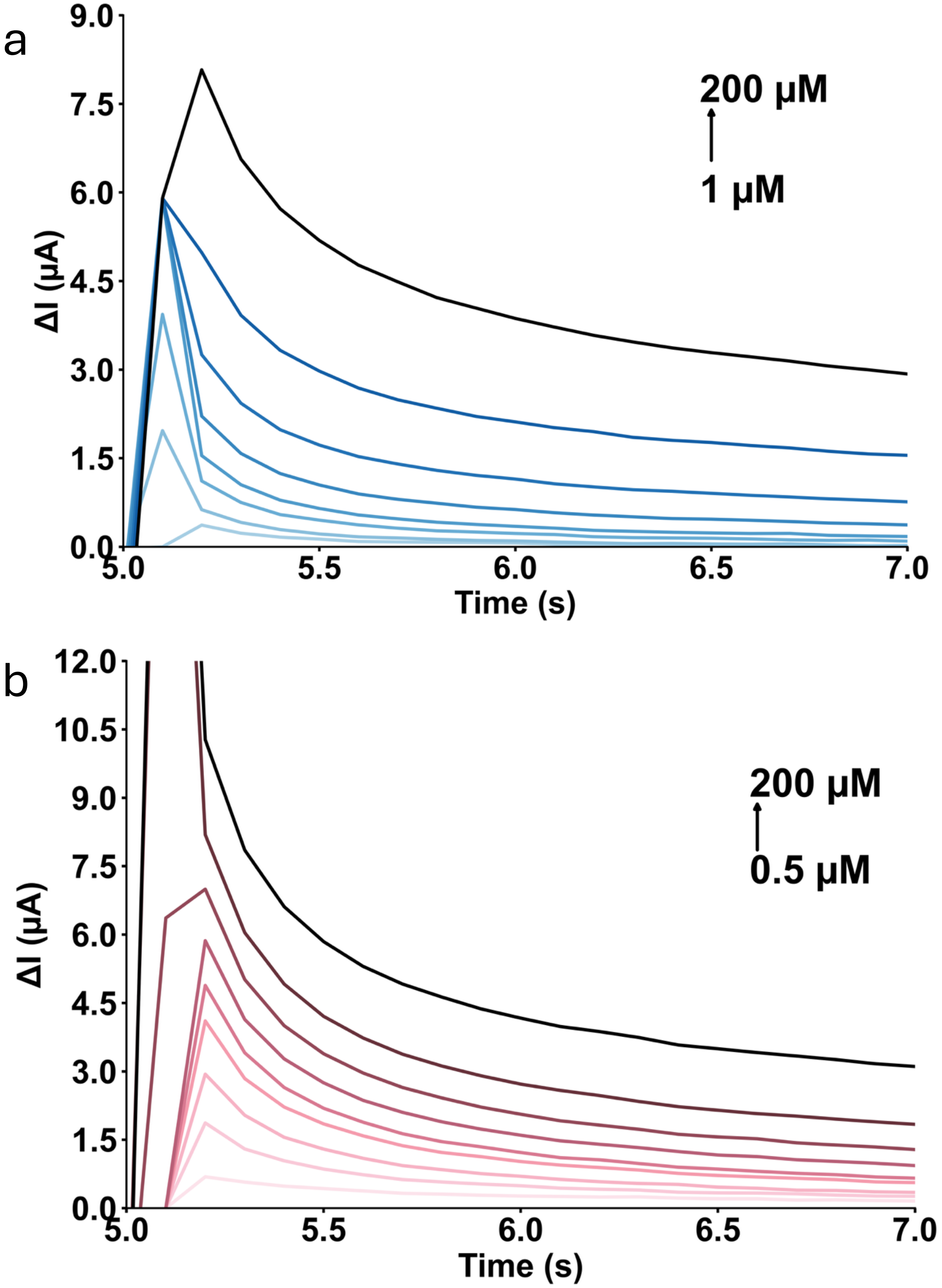

Figure S9. Baseline corrected CAs of a) Au@Pt/SWCNTs 1:3, and b) Au@Pt/SWCNTs 5:3 vs. H_2O_2 1-200 and 0.5-200 µM/ PBS, respectiveley, Scan rate: 50 mV s^{-1}.

Table S4: Averaged atomic percentages of carbon (C), nitrogen (N), and oxygen (O) obtained from XPS measurements of BSA-incubated SWCNT and Au@Pt NRs/SWCNT platforms (mean ± SD, N = 5), spot size: 400 µm.

**Sample**	**C (%)**	**N (%)**	**O (%)**	**N / C ratio**
Pristine SWCNTs	77.8 ± 4.1	8.4 ± 2.5	13.8 ± 1.6	0.108
Au@Pt/SWCNTs 1:3	77.0 ± 1.9	8.8 ± 1.6	14.2 ± 1.1	0.114
Au@Pt/SWCNTs 5:3	71.4 ± 1.1	12.4 ± 1.3	16.2 ± 0.4	0.174

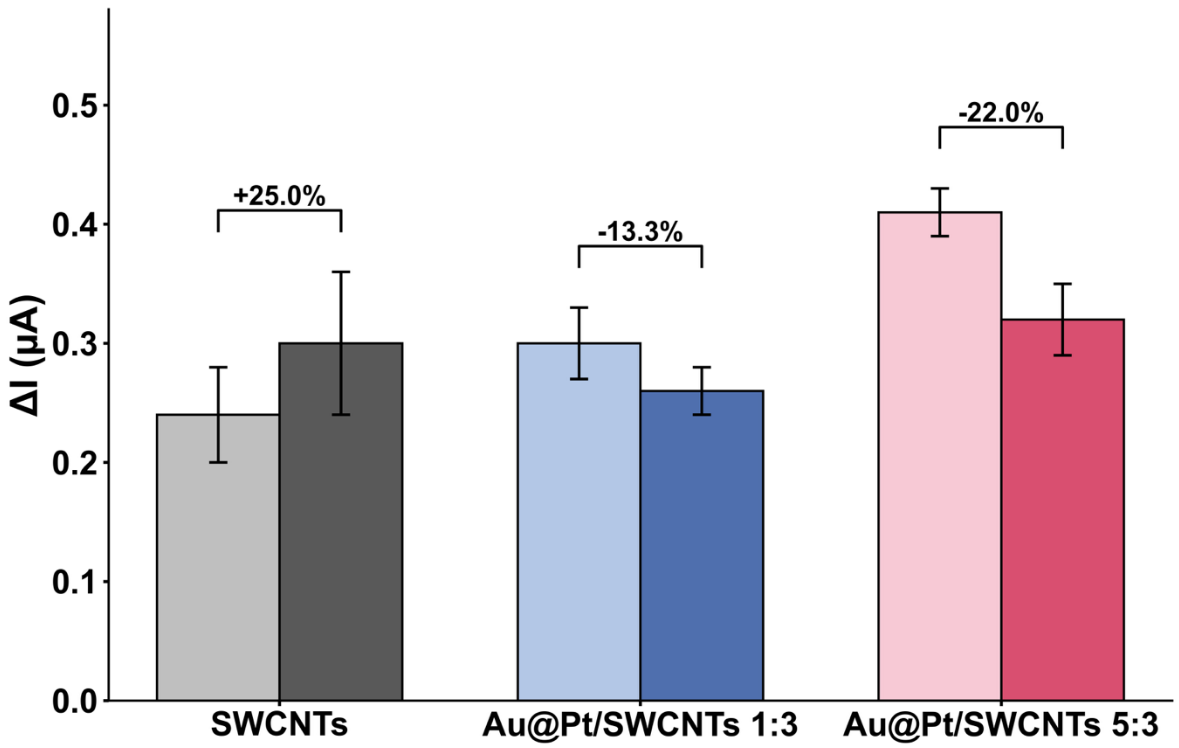

Figure S10. Bar chart showing the baseline-corrected anodic CV peak current difference for 10 μM DA before (light) and after (dark) BSA incubation on pristine SWCNTs (gray), Au@Pt/SWCNTs 1:3 (blue), and Au@Pt/SWCNTs 5:3 (pink).

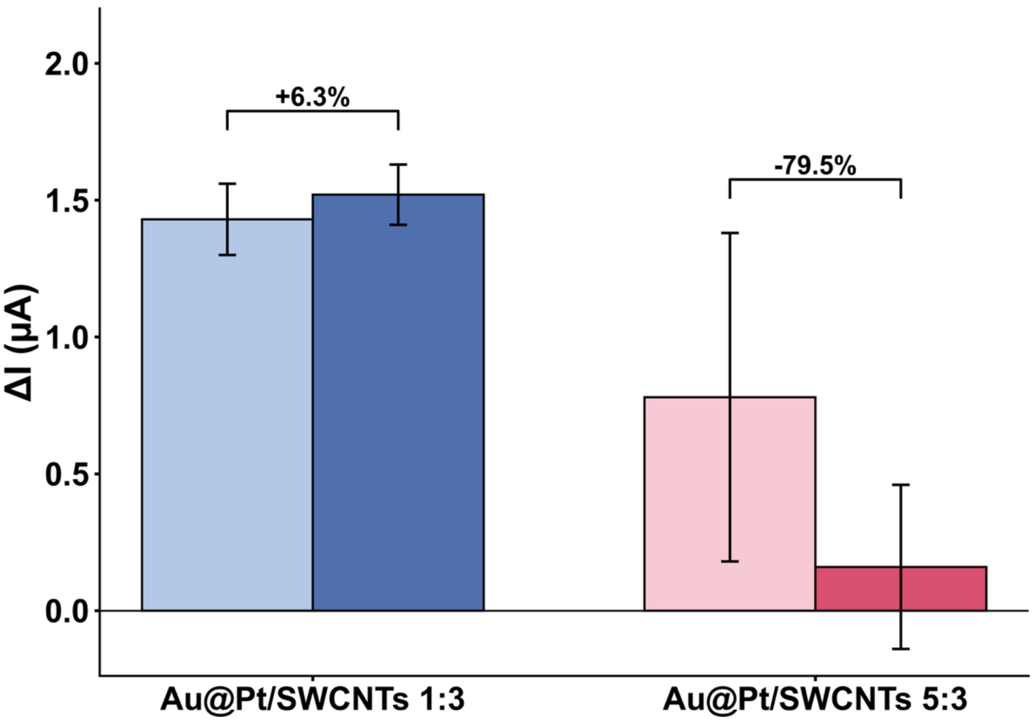

Figure S11. Comparison of baseline-corrected CA current responses to 50 μM H_2O_2 pre- (light) and post- (dark) BSA incubation for Au@Pt/SWCNTs 1:3 (blue), and Au@Pt/SWCNTs 5:3 (pink).

Table S5: Peak current values for the pre- and post- BSA incubations of Pristine and modified electrodes vs. DA and H_2O_2.

Sample	DA		H_2O_2	
	Pre-BSA Ipa (μA)	Post-BSA Ipa (μA)	Pre-BSA Ipa (μA)	Post-BSA Ipa (μA)
Pristine SWCNTs	0.24 ± 0.04	0.30 ± 0.06	-	-
Au@Pt/SWCNTs 1:3	0.30 ± 0.03	0.26 ± 0.02	1.43 ± 0.13	1.52 ± 0.11
Au@Pt/SWCNTs 5:3	0.41 ± 0.02	0.32 ± 0.03	0.78 ± 0.6	0.16 ± 0.03

Table S6: Comparison Table for electrochemical sensors with focus on DA detection. In some studies the sensitivity was given in respect to current and not currents density. This is indicated by /A in the table.

**Electrode materials**	**Linear range (µM)**	**LOD (µM)**	**Sensitivity (µA µM^{-1} cm^{-2})**	**Ref**
MWCNT-EDAS-AuNPs	10^{-4} - 8 × 10^{-3}	0.08	0.03/A	1
AuNPs/ MWCNTs	0.4 - 5.7	0.07	2.06/A	2
MWCNT@PDOP@PtNPs	0.25 - 20	0.08	1.03/A	3
AuNBP/MWCNTs	0.05 - 2700	0.015	N.A.	4
Au@NAC-MWCNTs	0.1 - 250	0.03	N.A.	5
Pt CNC/MWCNT@GO/GCE	0.8 - 300	0.27	N.A.	6
SWCNTs	1 - 200	0.02	0.352	**This work**
Au@Pt SWCNTs 1:3	1 - 200	0.40	0.384	**This work**
Au@Pt SWCNTs 5:3	1 - 200	0.62	0.536	**This work**

MWCNT-EDAS-AuNPs: Multiwalled carbon nanotube -silicate molecule *N*-[3(trimethoxysilyl)propyl]ethylenediamine-gold nanoparticles; MWCNT@PDOP@PtNPs: Multiwalled carbon nanotube@polydopamine@platinum nanoparticles; AuNBP/MWCNTs: Gold nanobipyramid/multi-walled carbon nanotube; Au@NAC-MWCNTs: Gold@ N-acetyl-l-cystein - multiwalled carbon nanotube; Pt CNC/MWCNT@GO/GCE: platinum concave nanocube/ multiwalled carbon nanotube@ graphene oxide/ glassy carbon electrode.

Table S7: Comparison Table for electrochemical sensors with focus on H_2O_2 detection. In some studies the sensitivity was given in respect to current and not currents density. This is indicated by /A in the table

Electrode materials	Linear range (µM)	LOD (µM)	Sensitivity ($\mu A\ \mu M^{-1}\ cm^{-2}$)	Ref
Pt-CNFs	1- 100<br>100 -1000	0.21	0.257	7
Pt NP/MWCNT/Au SPE NF	5 - 2000	1.23	0.003359/A	8
Au/Pt NP-coated CNT/silica nanocables	0.5 - 1670	0.3	N.A.	9
Pt-MWCNTs-IL	0.25 -7000	0.25	2.4	10
AuNPs/MWCNT/PANI/Au	3 - 600	0.3	3300/A	11
Au@Pt NRs/ GCE	05 - 50	0.189	0.368	12
Au@Pt SWCNTs 1:3	1 - 5<br>5 - 200	0.127	1.138	**This work**
Au@Pt SWCNTs 5:3	0.5 - 5<br>5 - 200	0.045	7.973	**This work**

Pt-CNFs: Platinum-carbon nanofibers; Pt NP/MWCNT/Au SPE NF: Platinum nanoparticle-decorated carbon nanotube clusters on screen-printed gold nanofilm; Pt-MWCNTs-IL: Platinum-decorated multiwalled carbon nanotubes-ionic liquid composite; AuNPs/MWCNT/PANI/Au: Polyaniline/ multiwalled carbon nanotubes/ gold nanoparticles/ gold electrode; Au@Pt NRs/ GCE: gold platinum coreshell nanorods/ glassy carbon electrode.

References

1. Vinoth, V., Wu, J. J., Asiri, A. M. & Anandan, S. Simultaneous detection of dopamine and ascorbic acid using silicate network interlinked gold nanoparticles and multi-walled carbon nanotubes. *Sens. Actuators B Chem.* **210**, 731–741 (2015).
2. Caetano, F. R., Felippe, L. B., Zarbin, A. J. G., Bergamini, M. F. & Marcolino-Junior, L. H. Gold nanoparticles supported on multi-walled carbon nanotubes produced by biphasic modified method and dopamine sensing application. *Sens. Actuators B Chem.* **243**, 43–50 (2017).
3. Lin, M. *et al.* High loading of uniformly dispersed Pt nanoparticles on polydopamine coated carbon nanotubes and its application in simultaneous determination of dopamine and uric acid. *Nanotechnology* **24**, 065501 (2013).
4. Cheng, J. *et al.* A novel electrochemical sensing platform for detection of dopamine based on gold nanobipyramid/multi-walled carbon nanotube hybrids. *Anal. Bioanal. Chem.* **412**, 2433–2441 (2020).
5. Wang, Z. *et al.* Simultaneous and selective measurement of dopamine and uric acid using glassy carbon electrodes modified with a complex of gold nanoparticles and multiwall carbon nanotubes. *Sens. Actuators B Chem.* **255**, 2069–2077 (2018).
6. Zhang, X. & Zheng, J. High-index {hk0} facets platinum concave nanocubes loaded on multiwall carbon nanotubes and graphene oxide nanocomposite for highly sensitive simultaneous detection of dopamine and uric acid. *Talanta* **207**, 120296 (2020).
7. Isoaho, N. *et al.* Pt-grown carbon nanofibers for detection of hydrogen peroxide. *RSC Adv.* **8**, 12742–12751 (2018).
8. Niu, X., Zhao, H., Chen, C. & Lan, M. Platinum nanoparticle-decorated carbon nanotube clusters on screen-printed gold nanofilm electrode for enhanced electrocatalytic reduction of hydrogen peroxide. *Electrochimica Acta* **65**, 97–103 (2012).
9. Guo, S. *et al.* Carbon Nanotube/Silica Coaxial Nanocable as a Three-Dimensional Support for Loading Diverse Ultra-High-Density Metal Nanostructures: Facile Preparation and Use as Enhanced Materials for Electrochemical Devices and SERS. *Chem. Mater.* **21**, 2247–2257 (2009).
10. Joshi, V. S., Kreth, J. & Koley, D. Pt-Decorated MWCNTs–Ionic Liquid Composite-Based Hydrogen Peroxide Sensor To Study Microbial Metabolism Using Scanning Electrochemical Microscopy. *Anal. Chem.* **89**, 7709–7718 (2017).
11. Narang, J., Chauhan, N. & Pundir, C. S. A non-enzymatic sensor for hydrogen peroxide based on polyaniline, multiwalled carbon nanotubes and gold nanoparticles modified Au electrode. *The Analyst* **136**, 4460 (2011).
12. Mostafiz, B. *et al.* Various Configurations of Au@Pt Nanostructures on Modified Electrochemical Sensors for H_2 O_2 Detection. *ACS Appl. Nano Mater.* **8**, 15382–15394 (2025).
